# *HIPSTR* and thousands of lncRNAs are heterogeneously expressed in human embryos, primordial germ cells and stable cell lines

**DOI:** 10.1038/srep32753

**Published:** 2016-09-08

**Authors:** Dinar Yunusov, Leticia Anderson, Lucas Ferreira DaSilva, Joanna Wysocka, Toshihiko Ezashi, R. Michael Roberts, Sergio Verjovski-Almeida

**Affiliations:** 1 Departamento de Bioquímica, Instituto de Química, Universidade de São Paulo, 05508-000 São Paulo, SP, Brazil; 2 Instituto Butantan, 05503-900 São Paulo, SP, Brazil; 3 Department of Chemical and Systems Biology and Department of Developmental Biology, Stanford University School of Medicine, Stanford, CA 94305, USA; 4 Division of Animal Sciences, University of Missouri, Columbia, MO 65211, USA; 5 Department of Biochemistry, University of Missouri, Columbia, MO 65211, USA

## Abstract

Eukaryotic genomes are transcribed into numerous regulatory long non-coding RNAs (lncRNAs). Compared to mRNAs, lncRNAs display higher developmental stage-, tissue-, and cell-subtype-specificity of expression, and are generally less abundant in a population of cells. Despite the progress in single-cell-focused research, the origins of low population-level expression of lncRNAs in homogeneous populations of cells are poorly understood. Here, we identify *HIPSTR* (Heterogeneously expressed from the Intronic Plus Strand of the TFAP2A-locus RNA), a novel lncRNA gene in the developmentally regulated *TFAP2A* locus. *HIPSTR* has evolutionarily conserved expression patterns, its promoter is most active in undifferentiated cells, and depletion of *HIPSTR* in HEK293 and in pluripotent H1_BP_ cells predominantly affects the genes involved in early organismal development and cell differentiation. Most importantly, we find that *HIPSTR* is specifically induced and heterogeneously expressed in the 8-cell-stage human embryos during the major wave of embryonic genome activation. We systematically explore the phenomenon of cell-to-cell variation of gene expression and link it to low population-level expression of lncRNAs, showing that, similar to *HIPSTR*, the expression of thousands of lncRNAs is more highly heterogeneous than the expression of mRNAs in the individual, otherwise indistinguishable cells of totipotent human embryos, primordial germ cells, and stable cell lines.

Eukaryotic genomes are pervasively transcribed^1^,^2^,^3^, producing thousands of uncharacterized transcripts, the majority of which are classified as long non-coding RNAs (lncRNAs) (ref. 4). LncRNAs are simply defined as long (>200 nt) non-protein coding transcripts, and as such they represent a very broad, widely uncharacterized group that includes non-functional transcripts resulting from transcriptional noise (random transcription initiation by RNA Pol II throughout the genome), and lncRNAs exerting their function either passively through the act of their transcription or actively in *cis* and in *trans*^5^. When compared to mRNAs, lncRNAs are expressed at lower levels with considerably higher tissue-specificity^6^, developmental stage-specificity^7^, and in heterogeneous tissues – also cell-subtype specificity^8^. It has been proposed that low expression levels of lncRNAs originate from high cell-to-cell variation in expression of lncRNA genes^9^. Nonetheless, arguing with this hypothesis and findings from mouse bone-marrow-derived dendritic cells^10^, a recent study exploited single-cell RNA-FISH and demonstrated that cell-to-cell variation of lncRNAs expression is similar to that of mRNAs^11^. Overall, the relationship of the low population-level expression of lncRNAs and cell-to-cell variation of gene expression remains largely unexplored.

Although, antisense transcription has been proposed to occur in 74% of human gene loci^12^, antisense lncRNAs remain the least studied group of all lncRNAs. Five prime regions of antisense lncRNA genes coincide with the presence of promoter-associated chromatin marks, CpG islands, and RNA Pol II binding^13^,^14^, and 28% of antisense transcripts were detected in the absence of expression of their overlapping genes^15^, further supporting the independence of these transcription units. Our previous work demonstrates that such antisense transcription units frequently produce monoexonic lncRNAs^16^. The most highly expressed antisense lncRNAs are transcribed antisense to genes encoding transcription regulators^12^. The importance of antisense lncRNAs^17^ is illustrated by a particular example of *ANRASSF1* (ref. 18) oncogenic lncRNA, and by differential expression of multiple antisense lncRNAs in pancreatic cancer^13^, and renal cell carcinoma^14^, where the expression of antisense lncRNAs is correlated with expression of their sense counterparts^12^,^14^,^16^. The widely accepted assumption that a large portion of antisense lncRNAs regulates their overlapping genes^19^ might nonetheless be a poor predictor of function for any yet uncharacterized antisense lncRNA.

In this study, we identified a novel lncRNA, which we named *HIPSTR*, that is expressed from the opposite strand of *TFAP2A*, the gene encoding a transcription factor (TF) involved in tumorigenesis^20^, and important for neural crest^21^,^22^,^23^,^24^ and trophectoderm^25^,^26^ development. We found that *HIPSTR* has conserved expression patterns between human and mouse, and that its promoter demarcation is conserved in the amniotes. Unlike previously characterized antisense lncRNAs, we found that *HIPSTR* expression levels do not correlate with the expression of its overlapping *TFAP2A* gene in cell lines, tissues, and developmental models. Silencing of *HIPSTR* led to differential expression of a group of genes involved in development and differentiation. Consistently, we show that *HIPSTR* is activated independently from *TFAP2A* during early development, where it has heterogeneous expression, being expressed in only a subset of cells within totipotent human embryos. We further explore the phenomenon of heterogeneous expression and demonstrate that lncRNAs in totipotent human embryos, human embryonic stem cells (hESCs), human primordial germ cells (hPGCs), and myelogenous leukemia cells (K562) have significantly higher cell-to-cell variation in expression than mRNAs.

## Results

### *HIPSTR* is a *bona fide* antisense lncRNA with evolutionarily conserved expression patterns

**Figure 1:**
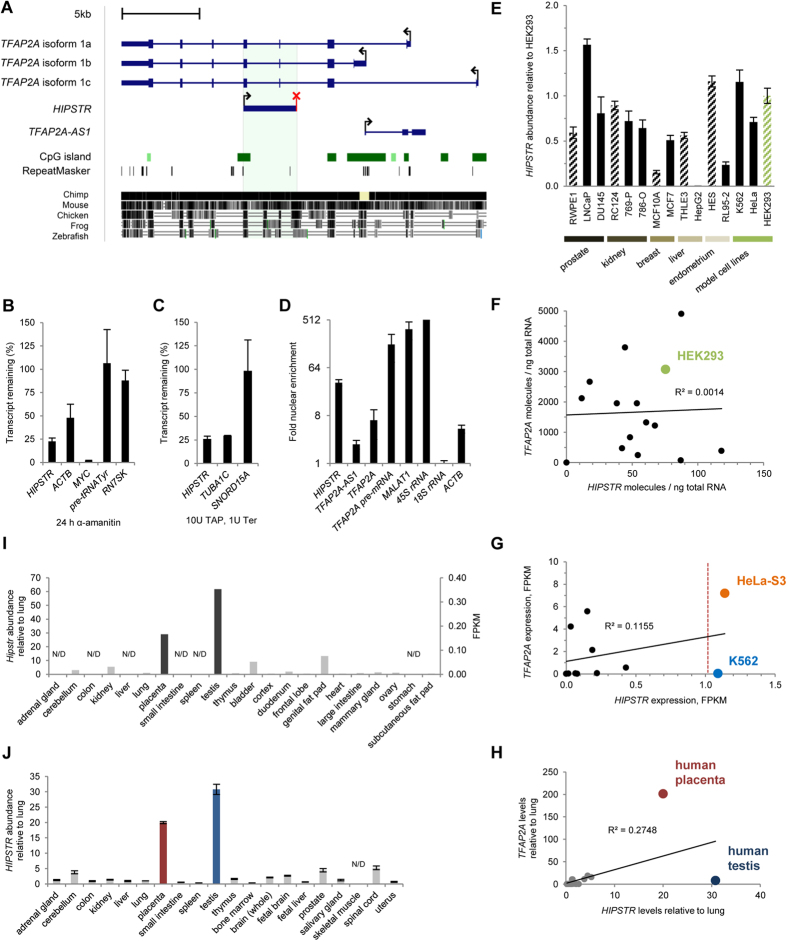
*HIPSTR* is a *bona fide* lncRNA. (**A**) Genomic position of human *HIPSTR* relative to the *TFAP2A* locus genes. The predicted *HIPSTR* polyadenylation signal is marked with a red “X” sign; genomic coordinates of the region shown are hg19 chr6:10396400–10420700. (**B**) RNA Pol II inhibition by α-amanitin in HeLa cells decreases *HIPSTR* levels; known RNA Pol II transcripts (*ACTB, MYC*) and RNA Pol III transcripts (pre-tRNA^Tyr^, *7SK*) served as controls. (**C**) 5′-cap structure removal by co-treatment of HeLa cells total RNA with Terminator 5′-phosphate-dependent exonuclease (Ter) and tobacco acid pyrophosphatase (TAP) reduces levels of *HIPSTR*; capped *TUBA1C* and uncapped *SNORD15A* transcripts served as controls. (**D**) HeLa cells fractionation shows nuclear enrichment of *HIPSTR*; nuclear enrichment of *TFAP2A* and *TFAP2A-AS1* is comparable with that of *ACTB; TFAP2A* pre-mRNA, *MALAT1* lncRNA, and 45S rRNA served as nuclear fraction controls; 18S rRNA served as cytoplasmic fraction control. The same RNA samples were used as in ref. 69, and data shown on (**B**–**D**) for control transcripts are the same as presented on Fig. 3A,B,D in ref. 69. (**E**) *HIPSTR* expression cannot be associated with tumor or non-tumor phenotype, as measured in human tumor (solid bars) and non-tumor (hatched bars) cell lines; expression in non-tumor HEK293 cell line (hatched green bar) is shown for comparison. (**F**) *HIPSTR* expression does not correlate with *TFAP2A* levels in the human cell lines shown on (**E**). (**G**) *HIPSTR* expression does not correlate with *TFAP2A* levels in the ENCODE Project^29^ RNA-seq data from human cell lines (A549, GM12878, H1 hESCs, HeLa-S3, HepG2, HMEC, HSMM, HUVEC, K562, MCF7, NHEK). (**H**) *HIPSTR* expression does not correlate with *TFAP2A* levels in the human tissues shown on (**J)** (see below). (**I**) Mouse *Hipstr* (mm9 chr13:40818458–40821725) ortholog expression across a panel of mouse tissue RNA samples from the Mouse ENCODE Project^70^ RNA-seq data. (**J**) *HIPSTR* expression across a panel of human tissue RNA samples. Data shown on (**B**–**F**,**H**,**J**) are RT-qPCR read-outs of three independent experiments, error bars represent SD; data on (**I**,**G**) is our re-analysis of public RNA-seq; N/D – not detected.

Aiming at the identification of novel antisense lncRNAs possibly associated with prostate cancer, we obtained strand-specific deep RNA-seq data from LNCaP prostate cancer cell line and searched for antisense transcription events in loci encoding TFs. *TFAP2A* encodes a TF known to be involved in various cancers (reviewed in ref. 20), including prostate cancer^27^,^28^, where *TFAP2A* is downregulated, and its promoter is hypermethylated in LNCaP and DU145 model cell lines, as well as in clinical samples^27^. We focused on a putative monoexonic antisense lncRNA gene located between exons 2 and 5 of *TFAP2A* on the opposite genomic strand (Fig. 1A). We later named this lncRNA gene *HIPSTR* (Heterogeneously expressed from the Intronic Plus Strand of the TFAP2A-locus RNA).

We combined our results of RACE PCR (Fig. S1A), with our and public RNA-seq data to obtain the full-length sequence of the unspliced polyadenylated *HIPSTR* lncRNA (3427 nt, chr6:10404735–10408161 in human genome assembly hg19; Fig. 1A). Analysis of ENCODE Project data^29^ showed that *HIPSTR* has an alternative TSS in HeLa-S3 cells, located more than 600 bp upstream of the *HIPSTR* TSS in LNCaP or K562 cells (Fig. S1A). It remains to be investigated whether this alternative *HIPSTR* isoform is functionally different from the *HIPSTR* isoform described in this study. It is also evident from RNA-seq data that *HIPSTR* transcripts are unspliced (Fig. 1A, S1A). *HIPSTR* TSS is located within an 818-bp-long CpG island (Fig. 1A) and overlaps RNA Pol II ChIP-seq peaks from ENCODE Project data^29^ (Fig. S1B). We confirmed that *HIPSTR* is transcribed by RNA Pol II (Fig. 1B), and has a 5′-cap structure (Fig. 1C).

We next examined *HIPSTR* coding potential. First, we observed a strong nuclear enrichment of *HIPSTR* transcript (~33.5-fold, Fig. 1D), similar to some previously described regulatory nuclear lncRNAs (see Table 1 in ref. 30). In the nucleus, *HIPSTR* is associated with chromatin through the first 1000 nt of its sequence (Fig. S1C), although it is not possible to determine whether *HIPSTR* lncRNA remains associated with the chromatin at the same locus where it is produced. Both CPC (ref. 31) and CPAT (ref. 32) coding potential evaluation tools classified *HIPSTR* as non-coding. None of the potential ORFs within *HIPSTR* sequence showed any similarity to known proteins in a blastx search. There was no evidence of significant ribosome association with the *HIPSTR* sequence in the ribosome profiling data from ref. 33 (Fig. S1D). Finally, *in silico* analysis demonstrated that the longest potential ORF in the *HIPSTR* sequence (345 nt) can be expected to occur by chance in a 3427 nt-long transcript (Fig. S1E). Altogether, these data argue that *HIPSTR* is a *bona fide* lncRNA.

Considering the proposed roles for antisense RNAs in cancer^13^,^14^, we hypothesized that *HIPSTR* may be differentially expressed in tumor and non-tumor cell lines. We found that *HIPSTR* expression was not associated with tumor or non-tumor phenotype in prostate, kidney, breast, liver or endometrial cell lines (Fig. 1E). Moreover, *HIPSTR* expression did not correlate with its overlapping gene (*TFAP2A*) across the cell lines tested (Fig. 1F). The latter observation was further supported by analysis of *HIPSTR* and *TFAP2A* expression in ENCODE Project RNA-seq data sets^29^ (Fig. 1G), and in a panel of human tissue RNA samples (Fig. 1H). Consistent with previous reports^6^,^34^ for lncRNAs, we found that *HIPSTR* population-level expression was low and exceeded the value of 1 FPKM only in two (HeLa-S3 and K562) out of eleven ENCODE cell lines^29^ analyzed here (Fig. 1G).

We successfully detected *HIPSTR* transcription with RT-qPCR in a panel of mouse tissue RNA samples (Fig. S1F). Finally, we found that *HIPSTR* has an evolutionarily conserved tissue-specific expression pattern, and that it is predominantly expressed in testis and placenta of mouse (Mouse ENCODE Project RNA-seq data^29^ analyzed on Fig. 1I) and of human (Fig. 1J).

### *HIPSTR* promoter demarcation is conserved in the amniotes

The highest level of turnover among all classes of functional elements identified by the ENCODE Project^35^, and the lack of known orthologs in other species are common features of lncRNAs (reviewed in refs 36, 37). Only 19% of lncRNA families expressed in three or more tetrapod species studied by Necsulea *et al*.^38^ did originate more than 90 million years ago (Ma). In addition, only 21% of lncRNA loci that are present in human, chimpanzee, and macaque have an orthologous lncRNA outside of primates^38^. Interestingly, human lncRNAs transcribed from canonical RNA Pol II promoters emit strong and consistent signal of purifying selection, as opposed to lncRNAs transcribed from enhancers^39^. Notably, a characteristic promoter-associated H3K4me3 mark^40^,^41^ can be present on active and silent promoters^40^,^41^. We found that *HIPSTR* TSS was indeed demarcated by H3K4me3 (Fig. S2A) in K562 and NT2/D1 cells, which have high and undetectable levels of *HIPSTR*, respectively.

We first questioned the ability of DNA sequences surrounding *HIPSTR* TSS and occupied by H3K4me3 mark in K562 and NT2/D1 (ref. 29) to drive reporter gene transcription in four human cell lines (HeLa, HEK293, HepG2 and NT2/D1). We cloned sequences surrounding *HIPSTR* TSS (constructs pGL3-P1 to -P7, Fig. S2A) upstream of the firefly luciferase gene, and compared the luminescence signal produced by cells transfected with these reporters. We tested seven sequences, and they produced ~3- to ~903-fold stronger luminescence signal than negative control plasmid (pGL3-Basic) in the cell lines tested (Fig. S2B). Although we found that the endogenous *HIPSTR* gene is not expressed in NT2/D1 embryonal carcinoma cells, in this pluripotent cell line two *HIPSTR* promoter-luciferase constructs (pGL3-P1 and pGL3-P3) produced ~35–50-times stronger luminescence signal than did positive control construct (pGL3-SV40) (Fig. S2B). These data implies that specifically in pluripotent cells a strong positive regulator is present that would be able to drive transcription from *HIPSTR* promoter located on a plasmid. This is not the case for the endogenous *HIPSTR* promoter in NT2/D1 cells, likely due to the presence of H3K27me3 repressive chromatin mark in the *TFAP2A* locus (Fig. S2A). The latter explanation is further supported by the observation that the initial strong luminescence signal from *HIPSTR* promoter-luciferase constructs in NT2/D1 cells decreases as they lose pluripotency along the course of ATRA treatment (Fig. S2C).

Since *HIPSTR* expression patterns are conserved between human and mouse (Fig. 1I,J), we asked whether other mammalian species also have the *HIPSTR* gene. Since no deep strand-specific RNA-seq data sets are available for placenta and testis for organisms other than human and mouse, we hypothesized that the presence of a H3K4me3 mark may help to indirectly estimate the degree of *HIPSTR* promoter conservation and hence – of *HIPSTR* transcription unit itself.

**Figure 2:**
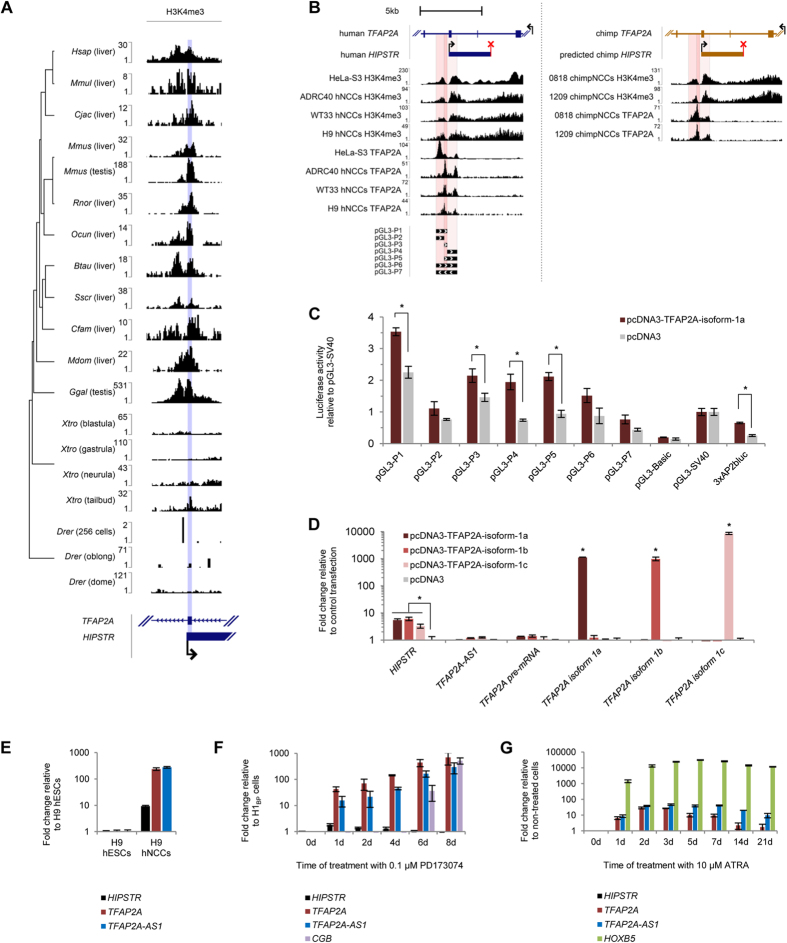
TFAP2A can regulate *HIPSTR* promoter, but *HIPSTR* and *TFAP2A* are not consistently co-induced in *in vitro* developmental models. (**A**) Re-analyses of H3K4me3 ChIP-seq data from refs 42, 43, 44, 45 reveal conserved *HIPSTR* promoter demarcation across the genomes of 10 mammalian species (see Methods) and of chicken, and absence of H3K4me3 mark around *HIPSTR* TSS orthologous region in frog and zebrafish. The maximal value on the y-axis scale corresponds to the highest H3K4me3 peak across the entire *TFAP2A* locus for each species. (**B**) Positions of the mapped H3K4me3 and TFAP2A ChIP-seq reads from ref. 29 (HeLa-S3 cells) and ref. 24 (three human NCC and two chimp NCC lines), and positions of the DNA sequences used for *HIPSTR* promoter-reporter assays (pGL3-P1 to -P7) relative to the *TFAP2A* locus genes. (**C**) Luciferase reporter assays in HEK293 cells upon TFAP2A isoform 1a overexpression. DNA sequences surrounding *HIPSTR* TSS (see above) were cloned upstream of the firefly luciferase gene, and co-transfected with plasmid overexpressing TFAP2A isoform 1a or with empty vector; pGL3-Basic served as negative control (no promoter upstream of the firefly luciferase); pGL3-SV40 served as positive control (SV40 promoter upstream of the firefly luciferase); 3xAP2bluc served as positive control for transactivation by TFAP2A isoform 1a. (**D**) Overexpression of TFAP2A isoforms 1a (dark red), 1b (red), or 1c (pink) upregulates endogenous *HIPSTR* in HEK293 cells, as measured with RT-qPCR. (**E**–**G**) *HIPSTR* is moderately co-upregulated with *TFAP2A* in *in vitro* derived human NCCs (**E**), weakly co-upregulated with *TFAP2A* in *in vitro* derived human TBCs (**F**), and not co-upregulated with *TFAP2A* in NT2/D1 cells treated with ATRA, where *HIPSTR* remains undetectable (**G**), as measured with RT-qPCR. Upregulation of *TFAP2A* gene itself (NCCs marker), of *CGB* (human TBCs marker), or *HOXB5* gene (induced by ATRA treatment in NT2/D1 cells)^47^ served as positive controls in the corresponding experiments shown on (**E**–**G**). Experiments presented on (**C**–**G**) were performed in triplicate, and error bars represent SD. For experiments on (**C**,**D**) the asterisks indicate statistical significance of the observed changes calculated with two-tailed t-test, equal variance (p-value < 0.01).

We analyzed public ChIP-seq data for various organisms^42^,^43^,^44^,^45^, and found H3K4me3 ChIP-seq peaks around *HIPSTR* TSS orthologous region in the samples of all 10 mammals tested, and of rooster, but not in any of the frog or zebrafish embryos (Fig. 2A). These results suggest that functional *HIPSTR* promoter demarcation existed approximately 325 Ma in a common ancestor of human and chicken^36^, and that therefore other amniotes likely have the *HIPSTR* gene.

### *HIPSTR* promoter can be stimulated by TFAP2A, but *HIPSTR* and *TFAP2A* are not consistently co-induced in developmental models *in vitro*

Increased activity of *HIPSTR* promoter in pluripotent cells suggests that *HIPSTR* may be involved in early embryonic development. Moreover, *HIPSTR* overlaps the developmentally-regulated *TFAP2A* gene that is induced and plays important roles in neural crest cells^21^,^22^,^23^,^24^ (NCCs), and in trophoblast cells^25^,^26^,^46^ (TBCs). Finally, *TFAP2A* expression can be transiently induced in human embryonal carcinoma NT2/D1 cells grown in the presence of ATRA (ref. 47). Interestingly, we found that TFAP2A ChIP-seq peaks were mapped to sequences upstream and downstream of *HIPSTR* TSS in HeLa-S3 cells (data from ref. 29), in human NCCs (data from ref. 23), and in the corresponding orthologous regions in chimp NCCs (data from refs 23, 24) (Fig. 2B). To assess the importance of TFAP2A in the regulation of *HIPSTR* promoter, we first overexpressed TFAP2A isoform 1a, and observed a significant increase in the luminescence signal from *HIPSTR* promoter-luciferase constructs in HEK293 (Fig. 2C), but not in HepG2 cells (Fig. S2D), while the activity of the TFAP2A-specific reporter (3xAP2bluc) increased in both experiments. To date, three TFAP2A isoforms with different functions were described^48^, and we next overexpressed each isoform in HEK293 and HepG2 cells (Fig. S2E), and measured expression of the endogenous *HIPSTR*. When overexpressed, each TFAP2A isoform upregulated endogenous *HIPSTR* in HEK293 cells (Fig. 2D), but was unable to initiate *HIPSTR* expression in HepG2 cells that lack endogenous *HIPSTR* expression (Fig. S2F). Aside from TFAP2A, dozens of TFs bind *HIPSTR* promoter, when assayed in different cell lines, as seen from ENCODE Project^29^ ChIP-seq data. Future studies should evaluate their relevance for *HIPSTR* promoter regulation in different cells.

Since *HIPSTR* gene is completely overlapped by developmentally regulated *TFAP2A* gene, and can be regulated by the protein product of the latter, we next hypothesized that both genes could be simultaneously induced during development. We induced *TFAP2A* expression *in vitro* in the three model systems mentioned above (hNCCs, hTBCs, and ATRA-treated NT2/D1 cells), and monitored *HIPSTR* expression levels. We confirmed a strong induction of *TFAP2A* transcription, and observed a simultaneous upregulation of divergently transcribed *TFAP2A-AS1* lncRNA in all three systems (Fig. 2E–G). However, >100-fold upregulation of *TFAP2A* was accompanied by only ~9.4-fold induction of *HIPSTR* in hNCCs (Fig. 2E) and by ~1.8-fold induction of *HIPSTR* in hTBCs (Fig. 2F). Moreover, ATRA treatment of NT2/D1 cells failed to induce *HIPSTR* expression (Fig. 2G). These results demonstrate the lack of consistent and robust co-induction of *HIPSTR* and *TFAP2A* in developmental models.

### *HIPSTR* silencing in HEK293 and H1_BP_ cells affects development-associated genes

**Figure 3:**
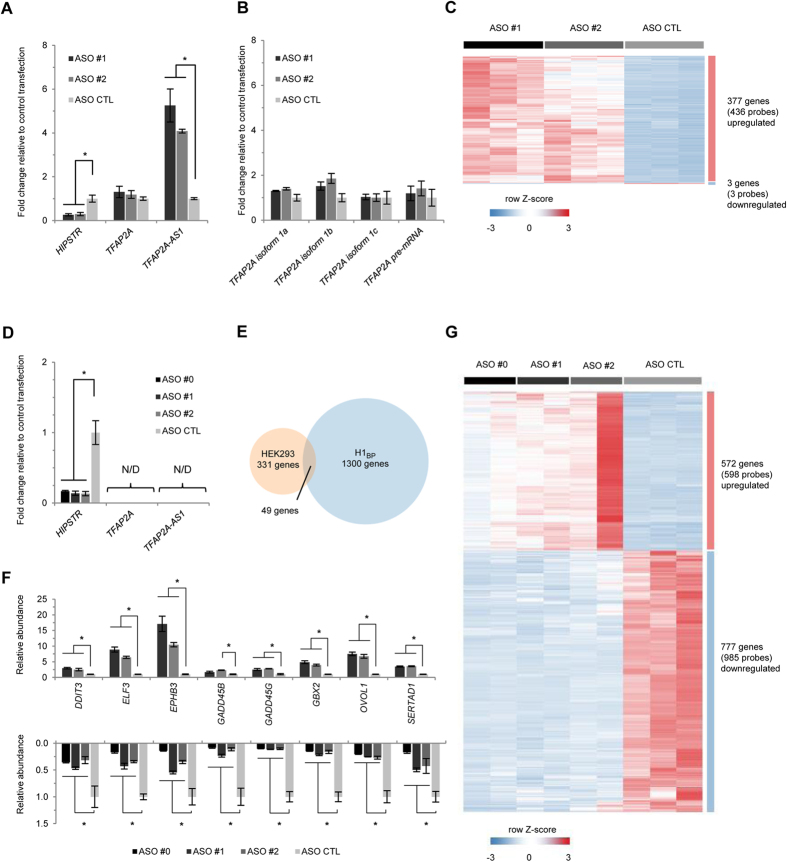
Developmental genes are affected by *HIPSTR* knockdown in HEK293 and H1_BP_ cells. (**A**) Effect of *HIPSTR* knockdown on the expression of *TFAP2A* locus genes in HEK293 cells. (**B**) *HIPSTR* knockdown does not significantly affect (p-value < 0.05, fold-change >2) the abundance of *TFAP2A* isoforms or pre-mRNA. (**C**) Heat map showing that *HIPSTR* knockdown in HEK293 cells leads to a significant upregulation of 377 annotated genes outside of *TFAP2A* locus (1% FDR, fold-change >2, also see Table S1). (**D**) Efficiency of *HIPSTR* knockdown in H1_BP_ cells. (**E**) Overlap between genes differentially expressed upon *HIPSTR* silencing in HEK293 and H1_BP_ cells (also see Table S2). (**F**) Validation of a group of genes, whose expression is significantly up- and downregulated by *HIPSTR* knockdown in HEK293 (top panel) and H1_BP_ cells (bottom panel), correspondingly. (**G**) Heat map demonstrating that *HIPSTR* knockdown in H1_BP_ cells leads to significant upregulation of 572 and downregulation of 777 genes (1% FDR, fold-change >2, also see Table S3). Data shown on (**A**,**B**,**D**,**F**) are RT-qPCR read-outs of three independent experiments, error bars represent SD; N/D – not detected; the asterisks indicate statistical significance of the expression differences (fold-change >2) calculated with two-tailed t-test, equal variance (p-value < 0.05).

*HIPSTR* levels do not correlate with the expression of *TFAP2A*. We then reasoned that chromatin-associated *HIPSTR* lncRNA might regulate other genes elsewhere in the genome in *trans*. Consistent with a relatively short half-life of this lncRNA (38 min, Fig. S3A), efficient *HIPSTR* silencing in HEK293 cells with a pool of targeting ASOs was achieved as early as 6 h after transfection (~71%, Fig. S3B), and the highest efficiency was reached 24 h post-transfection (~89%, Fig. S3B). *HIPSTR* silencing in HEK293 cells with each of the two targeting ASOs separately (ASO #1 and ASO #2; Fig. 3A) did not affect the overall levels of *TFAP2A* expression (Fig. 3A), but instead it significantly altered *TFAP2A-AS1* expression (Fig. 3A; see further details below). Neither the mRNA levels of specific *TFAP2A* isoforms, nor the levels of *TFAP2A* pre-mRNA were affected (Fig. 3B). *HIPSTR* knockdown resulted in genome-wide differential expression of 380 annotated genes (439 probes) located outside of the *TFAP2A* locus (Fig. 3C, Table S1), of which 377 (~99.2%) were upregulated. These results suggest a repressive function for *HIPSTR* in HEK293 cells. Accordingly, transient overexpression of *HIPSTR* in HEK293 cells (Fig. S3C) resulted in downregulation (Fig. S3D) of eight out of the twelve selected genes that were upregulated in *HIPSTR* knockdown experiments (Table S1). At the same time, Gene Ontology (GO) analysis of the genes upregulated by *HIPSTR* knockdown revealed their enrichment in “Developmental Process” and “Cell Differentiation” categories (Fig. S3E). A group of genes upregulated by *HIPSTR* knockdown in HEK293 cells was also upregulated by *HIPSTR* silencing in LNCaP cells (Fig. S3F), further validating these results.

Finally, we noted that in HEK293 cells, *HIPSTR* knockdown also did upregulate *TFAP2A-AS1* lncRNA (Fig. 3A). In turn, *TFAP2A-AS1* silencing with two different ASOs (ASO #3 and ASO #4; Fig. S3G) did upregulate *HIPSTR* (Fig. S3G) and several genes that were upregulated by *HIPSTR* knockdown (Fig. S3H, S3I). The upregulation of these genes (except for *ZSCAN10*) was inversely proportional to the extent of upregulation of *HIPSTR* upon *TFAP2A-AS1* knockdown with these ASOs (Fig. S3G, S3H), suggesting a partially overlapping function for these lncRNAs in HEK293 cells. Finally, TFAP2A protein levels in HEK293 cells were not affected by *HIPSTR* knockdown (Fig. S4A) and overexpression experiments (Fig. S4B).

We had established that *HIPSTR* promoter is most active in undifferentiated cells, but we could not induce *HIPSTR* expression with ATRA treatment of pluripotent carcinoma NT2/D1 cells. Conveniently, pluripotent H1_BP_ cells have a normal karyotype, express higher levels of *HIPSTR* than H1 hESCs from which they were derived (Fig. S4C), and have been proposed to be analogous to the outer cells of the 16-cell morula^49^. We silenced *HIPSTR* in H1_BP_ cells with three ASOs (ASO #1 and ASO #2 along with an additional ASO #0, Fig. 3D). *HIPSTR* knockdown did not change the morphology of H1_BP_ cells or expression of the core pluripotency network genes (Table S3). *TFAP2A* and *TFAP2A-AS1* remained undetectable (Fig. 3D), pointing at an independence of *HIPSTR* from *TFAP2A-AS1*, even though they may co-regulate a set of genes in HEK293 cells. Genome-wide, 49 annotated genes (53 probes) upregulated upon *HIPSTR* knockdown in HEK293 cells showed downregulation after *HIPSTR* depletion in H1_BP_ cells (Fig. 3E, Table S2), and we validated with RT-qPCR such an opposite differential expression pattern for a group of these genes after *HIPSTR* knockdown in HEK293 (Fig. 3F, top) and H1_BP_ cells (Fig. 3F, bottom). Overall, *HIPSTR* silencing in H1_BP_ cells resulted in 1349 significantly differentially expressed annotated genes (Fig. 3G, Table S3), of which 777 genes (985 probes; ~62.2%; Fig. 3G) were downregulated and showed enrichment in development- and metabolism-related GO categories (Fig. S4D). The remaining 572 genes (598 probes; ~37.8%; Fig. 3G) were upregulated and enriched for skin-, placenta-, lung-, and brain-specific expression (Fig. S4E). These results suggest that in the context of a pluripotent cell (H1_BP_ cells), *HIPSTR* is capable of both activating and repressing its target genes, whereas in a cell lacking pluripotency network associated factors (HEK293, LNCaP cells) *HIPSTR* acts solely as a repressor.

### *HIPSTR* expression in the early human embryo is restricted to a subset of cells

**Figure 4:**
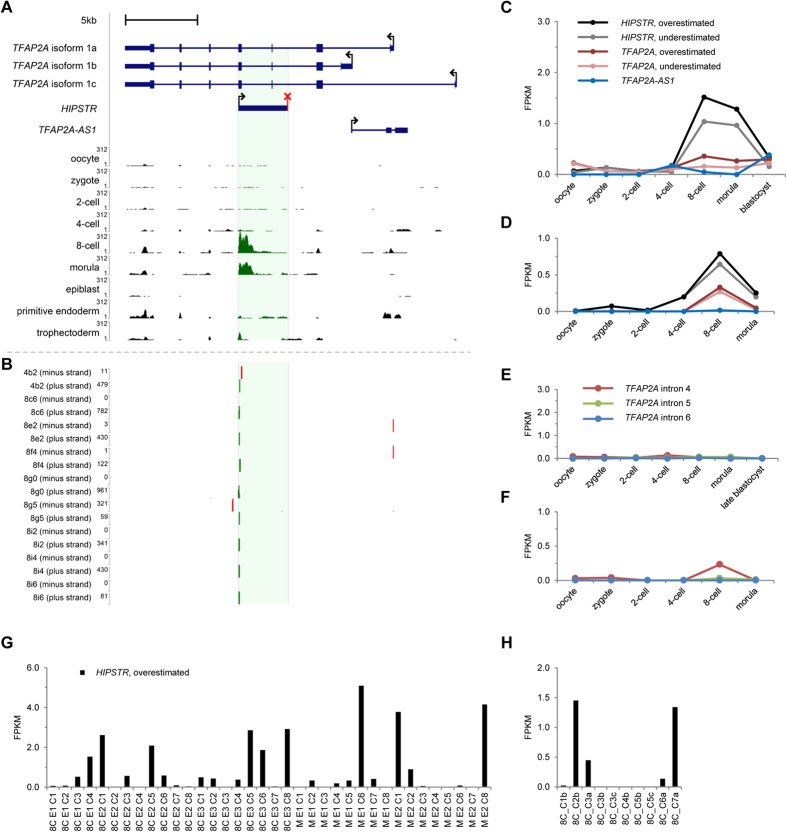
*HIPSTR* is expressed by a subset of cells in early human embryos. (**A**) Mapping of RNA-seq reads from ref. 7 illustrates specific expression of *HIPSTR*, and not *TFAP2A* or *TFAP2A-AS1*, in 8-cell and morula-stage human embryos. (**B**) Mapping of the 5′-ends of transcripts with strand-specific STRT-seq data from ref. 51 shows specific expression of *HIPSTR* in one cell (4b2) from a 4-cell human embryo, and in eight cells (8c6 through 8i6) originating from five different 8-cell human embryos; cell names are as in ref. 51. (**C**,**D**) Re-analyses of aggregate RNA-seq data for each developmental stage from ref. 7 on (**C**), and from ref. 50 on (**D**) confirms that *HIPSTR* induction in early embryos is independent from *TFAP2A* and *TFAP2A-AS1* genes. (**E**,**F**) *TFAP2A* pre-mRNA is not detectable in human oocytes and early embryos; re-analyses of aggregate RNA-seq data from ref. 7 on (**E**), and from ref. 50 on (**F**). (**G**,**H**) *HIPSTR* expression is restricted to a subset of cells in early human embryos, as inferred from RNA-seq data for 8-cell- and morula-stage embryos from ref. 7 in (**G**), or RNA-seq data for 8-cell-stage embryos from ref. 50 in (**H**); plotted are overestimated FPKM values for *HIPSTR* expression (see text).

We next addressed a possibility that *HIPSTR* induction occurs prior to and independent from the activation of *TFAP2A* in trophectoderm and/or neural crest during the course of embryonic development. In the past few years, several studies reported successful transcriptome sequencing of individual blastomeres of early human and mouse embryos^7^,^50^,^51^,^52^. We thus mapped (Fig. 4A) public RNA-seq data from two data sets^7^,^50^, and quantified *HIPSTR* expression in these data (Fig. 4C,D). As these RNA-seq data sets are not strand-specific, we present *HIPSTR* expression as “underestimated” and “overestimated” FPKM values, by accordingly excluding or including the reads mapping to exons of *TFAP2A* that overlap *HIPSTR*. We found that *HIPSTR*, and not *TFAP2A* or *TFAP2A-AS1* (Fig. 4C–F), is specifically upregulated in 8-cell and likely morula stage human embryos. We confirmed the presence of *HIPSTR* expression in 2–3 days-old human embryos (in one cell from a 4-cell stage embryo, and in eight cells from five separate 8-cell stage embryos) in the strand-specific single-cell-tagged reverse-transcription STRT-seq libraries from ref. 51 (Fig. 4B). These data indicate that *HIPSTR* gene is activated shortly after a major wave of human embryonic genome activation^7^ (EGA). It is evident from our analysis of public RNA-seq data from ref. 52 that expression of mouse *Hipstr* occurs in 2-cell embryos, soon after mouse EGA (Fig. S5B–S5D). Nonetheless, these latter observations are in conflict with mouse single-cell RNA-seq data from ref. 50, where we saw no evidence of expression in the *HIPSTR* orthologous region at all stages, including 2-cell stage (not shown). Hence, our results suggest that *HIPSTR* likely functions during the major wave of EGA in human embryos, but whether this is the case for mouse embryonic development remains an open question. Most intriguingly, we found that during the major wave of EGA within the 8-cell and morula stage human embryos (Fig. 4G,H), and in a population of K562 cells (Fig. S5A), *HIPSTR* expression is restricted to only a subset of cells.

### Cell-to-cell variability in expression of lncRNAs is higher than that of mRNAs

In a recent work, Cabili *et al*. used single-molecule RNA-FISH approach, and concluded that no difference exists in cell-to-cell variability in expression of mRNAs and lncRNAs^11^. This argues against a hypothesis that lncRNAs with low population-level abundance are instead expressed at high levels by a subset of cells within that population^9^. In agreement with the latter hypothesis are expression patterns of *HIPSTR* in early embryos and K562 cells, and of several mouse lncRNAs in bone-marrow-derived dendritic cells^10^.

**Figure 5:**
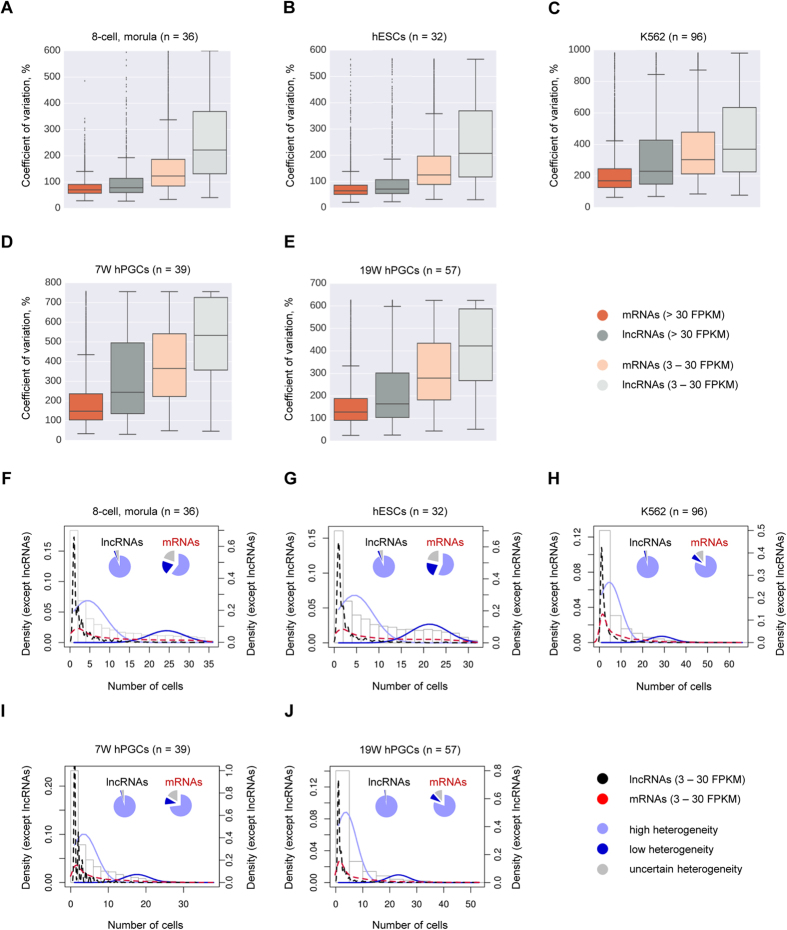
LncRNAs show higher heterogeneity of expression than mRNAs. (**A–E**) LncRNAs have higher cell-to-cell variation in expression than mRNAs. Coefficient of variation (CV) across all cells of a given single-cell RNA-seq data set was calculated for each expressed gene (>3 FPKM), and shown are box plots of CV values for highly expressed (>30 FPKM) mRNAs (dark orange) and lncRNAs (dark grey), and for moderately expressed (3–30 FPKM) mRNAs (light orange) and lncRNAs (light grey). Box shows the first and third interquartile range (IQR), the line inside the box shows the median, and whiskers encompass the CV values within 1.5 IQR below and above the first and third quartiles, respectively. Points outside the whiskers are CV outliers. All possible pairwise comparisons result in statistically significant differences, Welch’s t-test (p-value < 0.001). (**F**–**J**) Higher fraction of lncRNAs is classified as highly heterogeneously expressed, as compared to mRNAs. Plotted are density distributions of numbers of expressing cells calculated for lncRNAs (black dashed line), mRNAs (red dashed line), lncRNAs and mRNAs together (grey bars), and for modeled populations of genes with high (solid light blue line) or low (solid dark blue line) heterogeneity of expression. Pie charts demonstrate fractions of lncRNAs and mRNAs associated with the population of genes with high (light blue), low (dark blue) or uncertain (grey) heterogeneity of expression. Genes used for this analysis had expression >3 FPKM in at least one cell, and <30 FPKM in all cells of the corresponding data sets. Genes that contributed to the plots and pie charts on (**F**–**J**) were classified as belonging to either of the modeled populations of genes (with high or low expression heterogeneity) with a posterior probability >0.99, or were assigned the “uncertain heterogeneity” classification otherwise (posterior probability ≤0.99) (Tables S4,S5,S6,S7,S8). Single-cell RNA-seq data sets re-analyzed here were from: ref. 7 (8-cell and morula stage embryos, hESCs), ref. 53 (K562 cells), and ref. 54 (7W hPGCs and 19 W hPGCs). Number of individual cells used for each analysis is in parentheses in each panel heading.

To resolve this discrepancy between single-molecule RNA-FISH results and observations from single-cell RNA-seq data, we next systematically explored patterns of cell-to-cell expression variability of lncRNAs and mRNAs in human cells. For this, we used five single-cell RNA-seq data sets – from human totipotent blastomeres (36 cells; ref. 7), from pluripotent hESCs (32 cells; ref. 7), from K562 cells (96 cells; ref. 53), and from hPGCs of 7 weeks-old (7W; 39 cells; ref. 54) and of 19 weeks-old (19W; 57 cells, ref. 54) male embryos. We considered all expressed genes, defined here as those having max expression >3 FPKM (30-fold more stringent threshold than in refs 7, 54; see Methods) in at least one individual cell of a given data set, and compared the coefficient of variation of gene expression across the cells between lncRNAs and mRNAs. For genes with max expression within 3–30 FPKM, we saw a greater difference between non-coding and protein-coding transcripts than for those with max expression >30 FPKM (Fig. 5A–E). For the former group, the distribution of the numbers of cells was a mixture distribution. We fitted this mixture distribution with a finite mixture model with two populations, and used this model to classify lncRNAs and mRNAs as having high, low or uncertain heterogeneity of expression (Fig. 5F–J). For lncRNAs of this group (max expression 3–30 FPKM), only a small fraction showed low or uncertain (posterior probability <0.99) heterogeneity of expression −6.5%, 7.0%, 4.2%, 4.8%, and 2.3% in human totipotent blastomeres (Fig. 5F), hESCs (Fig. 5G), K562 cells (Fig. 5H), 7W hPGCs (Fig. 5I), and 19W hPGCs (Fig. 5J), respectively (Table S9). For example, in hESCs the known pluripotency regulator *TUNAR* (ref. 55) was assigned low heterogeneity flag in our analysis (Table S2). At the same time, *HIPSTR* was classified as a transcript with high heterogeneity of expression in 8-cell and morula-stage human embryos, and in K562 cells (Tables S4, S6), as expected. Remarkable heterogeneity of expression of lncRNAs was in a stark contrast to the much lower heterogeneity of expression of mRNAs with comparable expression levels (3–30 FPKM), of which 40%, 43%, 19%, 27%, and 20% were associated with low or uncertain heterogeneity of expression in human totipotent blastomeres, hESCs, K562 cells, 7W hPGCs, and 19W hPGCs, respectively (Fig. 5F–J; Table S9). Overall, lncRNAs analyzed here (max expression 3–30 FPKM) and assigned the high heterogeneity flag (H) constituted on average 74% of all expressed lncRNAs (>3 FPKM), while for mRNAs this fraction was only 35% (Table S8).

## Discussion

In the present work, we searched for novel antisense lncRNAs in the loci encoding TFs, and identified *HIPSTR* gene (Heterogeneously expressed from the Intronic Plus Strand of the TFAP2A-locus RNA) that is located on the opposite strand of *TFAP2A* gene. *HIPSTR* is transcribed by RNA Pol II into a capped, monoexonic, nuclear-enriched, chromatin-associated antisense lncRNA (Fig. 1A–D, S1A–S1D). *HIPSTR* is a *bona fide* antisense lncRNA; it is not associated with ribosomes and does not possess ORFs that could potentially encode any known polypeptide.

Many antisense transcripts were shown to regulate their overlapping or divergently transcribed genes (reviewed in ref. 56). We found that *HIPSTR* is not consistently co-induced with its overlapping and developmentally-regulated *TFAP2A* gene in *in vitro* developmental models, and that *HIPSTR* levels do not correlate with the expression of *TFAP2A* in cell lines and tissues. Accordingly, *HIPSTR* expression perturbations in HEK293 and H1_BP_ cells did not affect overall levels of *TFAP2A* mRNA (Fig. 3A,D), pre-mRNA (Fig. 3B), or TFAP2A protein levels (Fig. S4A). Genome-wide, *HIPSTR* likely acts context-dependently, as its knockdown upregulated a group of development-related genes (Fig. 3F, top) in non-pluripotent HEK293 cells, while in pluripotent H1_BP_ cells these genes were downregulated by *HIPSTR* silencing (Fig. 3F, bottom).

Since *HIPSTR* is capable of regulating developmental genes in different systems (Fig. S3F, S4D), and does not correlate with *TFAP2A*, we have searched public data for an evidence of *TFAP2A-*independent activation of *HIPSTR* during early development. We found that *HIPSTR* expression is indeed induced independently of *TFAP2A* specifically in 8-cell embryos, during the major wave of human EGA (Fig. 4A–D). Whether conservation of *HIPSTR* expression pattern (Fig. 1A,J) extends to the major wave of mouse EGA (2-cell stage) remains to be established, as existing RNA-seq data for early mouse embryos are inconsistent with respect to *Hipstr* expression (Fig. S5B–S5D). *Tfap2a-*null mice die perinatally with severe congenital defects^21^,^22^. Most interestingly, *Tfap2a*^−/−^ mice generated to date were obtained by targeting exons located upstream of the *Hipstr* gene and its promoter region. Should mouse *Hipstr* be induced in 2-cell embryos (and thus–prior to *Tfap2a* induction in trophectoderm or neural crest), genetic knockout studies would provide the ultimate evidence for the functional importance of *HIPSTR* during early embryonic development and the necessary support for mechanistic studies of *HIPSTR* function.

LncRNAs were proposed to function as modular scaffolds for chromatin modifying enzymes and TFs (ref. 57). Lower population-level expression of lncRNAs, as compared to mRNAs (refs 6, 34), might represent a serious obstacle for identification of partner proteins in RNA-Immunoprecipitation and endogenous RNA-pulldown assays, possibly resulting in false-negative results. We explore the origin of low population-levels of expression and show that lncRNAs are more heterogeneously expressed than mRNAs in individual, seemingly identical cells *in vitro* (Fig. 5A–C, Tables S4,S5,S6) and *in vivo* (Fig. 5D,E, Tables S7, S8). For example, in the K562 single-cell RNA-seq data set^53^, *HIPSTR* expression was completely absent (0 FPKM) from 73 individual cells, but reached 24.5 FPKM in 1 out of the 96 cells in that data set (Fig. S5A). This resulted in the population-average expression of *HIPSTR* of 0.91 FPKM in these 96 individual K562 cells, which was comparable with ENCODE Project bulk RNA-seq data for K562 cells (Fig. 1G).

The present study and the previously published analyses of transcriptomes of single mouse immune cells^10^ seem to be in disagreement with a recent work by Cabili *et al*.^11^, which shows that lncRNAs and mRNAs have a similar cell-to-cell abundance distribution. We found that 33 out of the 61 lncRNAs reported in ref. 11 were expressed (>3 FPKM) in at least one cell from at least one of the five single-cell RNA-seq data sets analyzed in the present study (Table S10). As anticipated, abundant lncRNAs, such as *GAS5* or *DANCR*, were expressed in the majority of the cells analyzed. On the other hand, in many instances, e.g. *lincFOXF1* or *lincMKLN1*, we found that lncRNAs assayed in Cabili *et al*.^11^ were as well expressed at relatively high levels, but only in few cells, and were classified as transcripts with high expression heterogeneity (Table S10). Similar pattern of expression was observed for another lncRNA, *linc-MUC20-1 (XLOC_024513*, not tested in Cabili *et al*.^11^). Heterogeneity of *linc-MUC20-1* expression was different in different cell types with comparable expression levels of this lncRNA: low expression heterogeneity in K562 cells (average – 2.88 FPKM, max – 19.91 FPKM, expressed in 27% of cells), and high heterogeneity – in 19 W hPGCs (average – 1.97 FPKM, max – 27.18 FPKM, expressed in 14% of cells). Expression pattern of *linc-MUC20-1* therefore demonstrates that expression heterogeneity of a given, readily detectable gene is not a fixed, but rather a very dynamic attribute that depends on a cell type considered for the analysis, which may explain the different conclusions reached by Cabili *et al*.^11^.

Based on these data, we conclude that highly heterogeneous expression in a population of seemingly identical cells is a common feature of human lncRNAs. This adds to the previously reported developmental stage, tissue, and cell subtype specificity of lncRNA expression^6^,^7^,^8^,^34^. It is important that the observed differences in cell-to-cell variation of abundance between lncRNAs and mRNAs cannot be explained by drop-out effects or technical noise of single-cell RNA-seq data, because our comparisons were done only for readily detectable genes (max expression >3 FPKM) with similar abundances within the same data sets.

Our results are of special importance for the studies of numerous recently identified and uncharacterized lncRNAs, as a complete absence of a given lncRNA in multiple cells in a population complicates statistical analyses, and high cell-to-cell variability in lncRNAs levels suggests that analyses of hundreds or even thousands of individual cells might be required to reveal meaningful expression correlations between heterogeneously expressed lncRNAs and other genes. For this, development of reliable and easy-to-use techniques facilitating enrichment for subpopulations of live cells expressing a lncRNA of interest will be required to uncover the exact mechanism of action of heterogeneously expressed lncRNAs, such as *HIPSTR*.

## Methods

Full methods are available on-line in the Supplementary Materials section.

### Antisense oligonucleotide (ASO)-mediated silencing

For ASO-mediated silencing of *HIPSTR* 4.5 × 10^5^ HEK293 cells or 2.4 × 10^5^ LNCaP cells were plated on 6-well plates 24 h before transfection. Transfections were performed by using 0.025 μl of Lipofectamine RNAiMAX (Invitrogen) per 1 pmol of transfected ASO. Transfection mixes were prepared in OptiMEM I Reduced Serum Medium (Gibco). *TFAP2A-AS1* silencing in HEK293 cells was done as described above for *HIPSTR*. To silence *HIPSTR* expression in H1_BP_ cells, 4 × 10^4^ cells were plated on 6-well plates 48 h before transfection, and cultured as described above; 0.013 μl of GenMute siRNA Transfection Reagent (SignaGen) per 1 pmol of ASO were used for transfection. Transfection mixes were prepared in 1x GenMute Transfection Buffer (SignaGen). A total of 300 pmol of ASO or mix of ASOs per well on 6-well plates was used for transfection. In all silencing experiments cells were collected for subsequent RNA or protein extraction 24 h after transfection with ASOs. For time-course *HIPSTR* knockdown assay in HEK293 cell line, cells were collected 6, 12, 24, 48, and 72 h after transfection with ASOs.

### Oligonucleotide sequences

All oligonucleotide sequences (primers and ASOs) are listed in Table S11.

### Microarray experiments

200 ng of total RNA from HEK293 cells or 100 ng of total RNA from H1_BP_ cells transfected with ASOs targeting *HIPSTR* were converted into Cy3- and Cy5-labeled cRNA with the Agilent Low Input Quick Amp Labeling Two Color Kit. Dye-swap technical replicates were created for each biological replicate. Three biological replicates of HEK293 cells transfected with each ASO were used for microarray experiments. In experiments with H1_BP_ cells, three biological replicates for control ASO, and two – for each of the targeting ASOs were assayed. Obtained cRNA samples were then hybridized to Agilent SurePrint G3 Gene Expression Microarrays (G4851B) 8 × 60 K as per manufacturer’s instructions. Data intensities were extracted from the slide images with Feature Extraction Software (Agilent Technologies) and normalized by using the Lowess method (Agilent Technologies).

All probes whose mean signal was lower than background on at least one array were filtered out. Signal intensities were normalized by 40% trimmed mean. Significance Analysis of Microarrays (SAM) with two-class comparison was then used to identify differentially expressed genes^58^. SAM q-value ≤ 0.01 and fold change ≥2 were considered as a threshold for identification of differentially expressed genes. Hierarchical clustering of differentially expressed genes was done with TIBCO Spotfire software by applying Z-score transformation of the normalized data intensities for each gene across all samples.

### Gene Ontology (GO) analysis

GO and tissue-specific expression analyses of annotated differentially expressed genes were performed with DAVID (https://david.ncifcrf.gov/) (ref. 59) with GOTERM_BP_ALL (or GOTERM_CC_FAT – for genes upregulated upon *HIPSTR* knockdown in H1_BP_ cells) and UP_TISSUE tables, respectively. Benjamini-Hochberg adjusted p-value ≤ 0.01 was used as a significance threshold. Genes are referred to as “annotated” if they have a HGNC symbol in Agilent annotation.

### RNA-seq and ChIP-seq analysis

ENCODE Project^29^ human long polyadenylated RNA-seq data for the indicated cell lines were obtained from GEO entry GSE30567, and mouse long RNA-seq – from GEO entry GSE36025. Ribosome profiling data from ref. 33 were downloaded from SRA entry SRA099816. K562 single-cell RNA-seq data were downloaded from SRA entry SRX495504 (ref. 53). Human and mouse embryonic single-cell RNA-seq data were retrieved from ENA entry PRJEB8994 (ref. 51), and from GEO entries GSE44183 (ref. 50), GSE36552 (ref. 7), GSE57249 (ref. 52), and GSE63818 (ref. 54). RNA-seq of DRB- (RNA Pol II elongation inhibitor) or vehicle-treated HEK293 cells from ref. 60 were obtained from GEO entry GSE66478. H3K4me3 ChIP-seq data for liver samples of 10 mammalian species were downloaded from Array Express website entry E-MTAB-2633 (ref. 44), for testis samples of mouse and rooster – from GEO entry GSE44588 (ref. 43), for frog blastula, gastrula, neurula and tailbud stage embryos – from GEO entry GSE41161 (ref. 42), and for zebrafish 256 cell, oblong and dome stage embryos – from GEO entry GSE44269 (ref. 45). TFAP2A and H3K4me3 ChIP-seq data for chimpanzee NCCs and hNCCs were obtained from GEO entry GSE70751 (ref. 24).

Sequencing data were preprocessed with Trimmomatic v.0.30 (ref. 61) with parameters *-phred33 LEADING:3 TRAILING:3 SLIDINGWINDOW:4:15*. Trimmomatic parameter *MINLEN:* was set at *16* for ChIP-seq reads, at *20* – for RNA-seq reads, except for RNA-seq data from ref. 33, for which it was set at *30*. Additional clipping of adapter sequence CTGTAGGCACCATCAAT was done for preprocessed RNA-seq reads from ref. 33 with fastx_clipper from FASTX Toolkit v.0.0.14 (http://hannonlab.cshl.edu/fastx_toolkit/). Human RNA-seq reads were mapped with TopHat v.2.0.12 (ref. 62) and Bowtie v.2.2.3 (ref. 63). The following parameters for TopHat were used: *–no-coverage-search –b2-sensitive*; for paired-end strand-specific RNA-seq data (except LNCaP RNA-seq), *–library-type fr-firststrand* parameter was used in addition to the mentioned above; for LNCaP RNA-seq data *–library-type fr-secondstrand* parameter was added. ChIP-seq reads were mapped by Bowtie v.2.2.3 with parameter: *–sensitive*. Read densities were retrieved with *genomecov* command from bedtools package v.2.20.1 (ref. 64), and UCSC Genome Browser tracks were built with bedGraphToBigWig v.4 (ref. 65). To count RNA-seq reads, TopHat paired-end RNA-seq data alignment output files were first sorted by read names with *sort* command from SAMtools package v. 0.1.19-44428cd (ref. 66). RNA-seq reads were counted with htseq-count v.0.6.1p1 (ref. 67), with parameter *-s yes* for single-end strand-specific data sets, *-s reverse* – for paired-end strand-specific data sets, and *-s no* for non-stranded data sets. For human data sets we used a GTF file from GSE57049 (ref. 11) complemented with the genomic coordinates of *HIPSTR*. Gene names in the output tables are those found in the abovementioned GTF file. To count mapped reads in mouse data sets, we used a GTF file for mouse genome assembly mm9 that was fetched from the illumina support site (https://support.illumina.com/). Gene expression levels were calculated in FPKM, considering gene length as a sum of all exonic non-overlapping sequences of all isoforms of a given gene. Unless stated otherwise, ChIP-seq and RNA-seq data are presented as aggregates of biological replicates for each indicated condition to increase resulting genome and transcriptome coverage, respectively.

To map RNA-seq and ChIP-seq data, the following reference genome assemblies were downloaded from UCSC Genome Browser (http://hgdownload.soe.ucsc.edu/downloads.html): galGal4 (chicken), panTro4 (chimpanzee), bosTau7 (cow), canFam3 (dog), xenTro3 (frog), hg19/GRCh37 (human), calJac3 (marmoset), mm9 (mouse), monDom5 (opossum), susScr3 (pig), oryCun2 (rabbit), rn5 (rat), rheMac3 (rhesus), danRer7 (zebrafish).

For single-cell RNA-seq data analyses, genes were considered as protein-coding if they were assigned RefSeq accession prefix NM_ (mRNA), or XM_ (mRNA predicted), or NP_ (peptide), or XP_ (peptide predicted); we also considered a gene as protein-coding in our analyses, if a possibility of read-through from a lncRNA gene to a protein-coding gene existed, according to GTF annotation file. Genes were considered as non-coding if they were assigned RefSeq accession prefix NR_ (ncRNA), or XR_ (ncRNA predicted), or were annotated as lincRNAs in the GTF file from ref. 11. For comparisons of expression profiles of non-coding and protein-coding genes in single cells, we considered only genes generating transcripts with total length of non-overlapping exonic sequences longer than 200 nt.

### Expression heterogeneity comparisons with the Finite Mixture model

To evaluate heterogeneity of gene expression in single cells, we used single-cell RNA-seq data sets for totipotent blastomeres from 8-cell and morula-stage human embryos, hESCs (both – from ref. 7), K562 cells (from ref. 53), or hPGCs from 7-week- or 19-week-old male embryos (both – from ref. 54). For each gene in each data set, we calculated the number of cells *N*, in which a given gene was expressed. The difference in cell-to-cell variability of gene expression was most evident between lncRNAs and mRNAs with max expression 3–30 FPKM in single cells, and thus we considered only genes with expression levels within this range, and did not consider genes whose expression was >30 FPKM in at least one cell of a data set under analysis. We counted a cell as *positive* for expression of a given gene if the expression level of that gene was >3 FPKM in that cell, which is a 30-fold more stringent cut-off than used in refs 7, 54.

We observed that, when assessed for all genes, the distribution of their corresponding *N* values is a mixture distribution. We used the *normalmixEM* function from mixtools v.1.0.4 R package^68^ to fit a model mixture distribution with two populations of genes – those with high or low heterogeneity of expression. Parameters used were: *number_of_components* = *2, lambda* = *0.5, sigma* = *0.5*.

We next applied the resultant model to calculate the posterior probability of each gene under analysis to belong to either the high or the low heterogeneity of expression population. If a given gene could be associated with one of the abovementioned populations with a posterior probability >0.99, it was assigned the “H” or “L” flag (for high or low heterogeneity of expression, respectively; Tables S4,S5,S6,S7,S8), otherwise the “U” (uncertain heterogeneity) flag was assigned.

## Additional Information

**Accession codes**: The microarray data sets supporting the results of this article were deposited in NCBI, and are available in the Gene Expression Omnibus (GEO) repository, GSE77937. RNA-seq data from LNCaP prostate cancer cell line were deposited in NCBI, and are available in the GEO repository, GSE79301. HIPSTR sequence is deposited in GenBank with accession number KU904338.

**How to cite this article**: Yunusov, D. *et al. HIPSTR* and thousands of lncRNAs are heterogeneously expressed in human embryos, primordial germ cells and stable cell lines. *Sci. Rep.* **6**, 32753; doi: 10.1038/srep32753 (2016).

## Supplementary Material

Supplementary Information

Supplementary Data Table S1

Supplementary Data Table S2

Supplementary Data Table S3

Supplementary Data Table S4

Supplementary Data Table S5

Supplementary Data Table S6

Supplementary Data Table S7

Supplementary Data Table S8

Supplementary Data Table S9

Supplementary Data Table S10

Supplementary Data Table S11
