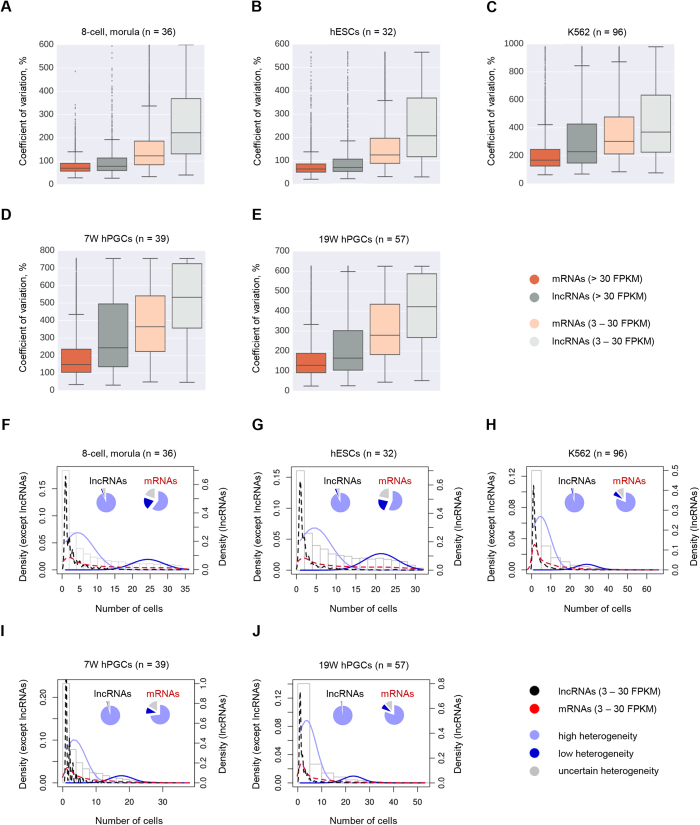# Corrigendum: *HIPSTR* and thousands of lncRNAs are heterogeneously expressed in human embryos, primordial germ cells and stable cell lines

**DOI:** 10.1038/srep44632

**Published:** 2017-03-16

**Authors:** Dinar Yunusov, Leticia Anderson, Lucas Ferreira DaSilva, Joanna Wysocka, Toshihiko Ezashi, R. Michael Roberts, Sergio Verjovski-Almeida

Scientific Reports 6: Article number: 3275310.1038/srep32753; published online: 09 08 2016; updated: 03 16 2017

This Article contains errors in Figure 5F–J, where the right-hand y axes ‘Density (lncRNAs)’ are incorrectly given as ‘Density (except lncRNAs)’. The correct Figure 5 appears below as Figure 1.

## Figures and Tables

**Figure 1:**