# Supplementary material for: HIPSTR and thousands of lncRNAs are heterogeneously expressed in human embryos, primordial germ cells and stable cell lines
Source: Sci Rep. 2016 Sep 8;6:32753. doi: 10.1038/srep32753 (PMC5015059; doi:10.1038/srep32753)
Supplement: Supplementary Information [file srep32753-s1.pdf]

HIPSTR and thousands of lncRNAs are heterogeneously expressed in human embryos, primordial germ cells and stable cell lines

Dinar Yunusov[1,2], Leticia Anderson[1,2], Lucas Ferreira da Silva[1,2], Joanna Wysocka[3], Toshihiko Ezashi[4], R. Michael Roberts[4,5], and Sergio Verjovski-Almeida[1,2]*

1 Departamento de Bioquímica, Instituto de Química, Universidade de São Paulo, 05508-000 São Paulo, SP, Brazil

2 Instituto Butantan, 05503-900 São Paulo, SP, Brazil

3 Department of Chemical and Systems Biology and Department of Developmental Biology, Stanford University School of Medicine, Stanford, CA 94305, USA

4 Division of Animal Sciences, University of Missouri, Columbia, MO 65211, USA

5 Department of Biochemistry, University of Missouri, Columbia, MO 65211, USA

*Corresponding author: sergio.verjovski@butantan.gov.br

Supplementary Methods

Cell culture

DU 145, 769-P, 786-O, MCF7, HepG2, NT2/D1, HEK293, HeLa (all – ATCC), RC-124 (CLS Cell Lines Service, GmbH) cell lines and HES human endometrial cells were cultured in DMEM medium (Vitrocell Embriolife) supplemented with 10 U/ml *Penicillin, 0.01 mg/ml Streptomycin* (1x Pen-Strep; Vitrocell Embriolife) and 10 % FBS (Vitrocell Embriolife). HES human endometrial cell line was a kind gift from Dr. Douglas Kniss (Ohio State University, Columbus, USA). H9 human embryonic stem cells (hESCs, WiCell) were cultured as described in ref. 1. $H1_{BP}$ cells were derived from H1 hESCs (WiCell) as described previously[2].

LNCaP and K562 cell lines (both – ATCC) were cultured in RPMI-1640 medium (Gibco) supplemented with 1x Pen-Strep (Vitrocell Embriolife) and 10 % FBS (Vitrocell Embriolife), and for LNCaP an additional 10 mM HEPES (Gibco). For RNA-seq experiments, LNCaP cells were grown in RPMI-1640 medium supplemented with 10 mM HEPES (Gibco) and 10 % charcoal stripped FBS (Sigma) for 48 h prior to RNA extraction.

RWPE-1 cells (ATCC) were cultured in K-SFM medium (Gibco) containing 0.05 mg/ml bovine pituitary extract (Gibco), 5 ng/ml EGF (Gibco), and 1x Pen-Strep (Vitrocell Embriolife).

MCF10A cells (ATCC) were cultured in DMEM/F12 medium (Vitrocell Embriolife) supplemented with 20 ng/ml EGF (Invitrogen), 10 μg/ml insulin (Invitrogen), 0.5 μg/ml hydrocortisone (Sigma), 0.1 μg/ml cholera toxin (Sigma), 1x Pen-Strep (Vitrocell Embriolife), and 5 % horse serum (Gibco).

RL95-2 cells (ATCC) were cultured in DMEM/F12 medium (Vitrocell Embriolife) supplemented with 1x Pen-Strep (Vitrocell Embriolife) and 10 % FBS (Vitrocell Embriolife).

THLE-3 cells (ATCC) were cultured on flasks precoated with FNC coating mix (AthenaES), and in BEGM medium (Clonetics) supplemented with 1x Pen-Strep (Vitrocell Embriolife), 10 % FBS (Vitrocell Embriolife), 5 ng/ml EGF (Invitrogen), 70 ng/ml Phosphoethanolamine (Sigma), supplemented with all additives from BEGM bullet kit (Clonetics), except for Epinephrine and Gentamycin/Amphotericin.

All cell lines were grown at 37 °C in 5 % CO_2-humidified atmosphere.

LNCaP RNA-seq

LNCaP RNA-seq libraries were prepared as described in ref. 3. In brief, LNCaP poly(A)$^+$ RNA was extracted with FastTrack MAG Maxi mRNA Isolation Kit (Invitrogen), as per manufacturer's protocol, treated with 25 U of DNase I, Amplification Grade (Invitrogen) for 1 h at room temperature, quantified with Quant-iT RiboGreen RNA Reagent (Invitrogen) and assessed for integrity on 2100 Bioanalyzer (Agilent). Obtained RNA samples were used for strand-specific paired-end RNA-seq library preparation, in accordance with the standard illumina protocol and two biological replicates were sequenced on a HiSeq 2000. Data were processed as described in the main text, and 298.37 million read pairs were successfully mapped to human genome assembly hg19.

5′ and 3′ rapid amplification of cDNA ends (RACE)

Human Prostate Marathon-Ready cDNA (Clontech) was used to validate strand-specific RNA-seq identification of *HIPSTR* in LNCaP prostate carcinoma cell line. The first round of the 5′ and 3′ RACE PCRs was done in complete agreement with Marathon-Ready cDNA library user manual (Clontech). The second round of RACE PCR was performed with nested strand-specific primers to increase the specificity of target product detection (Table S11). Obtained PCR products were gel-purified (Wizard SV Gel and PCR Clean-Up System; Promega), cloned into pGEM T-Easy vector (Promega), and sequenced.

***HIPSTR* coding potential analysis and polyadenylation signal prediction**

To assess *HIPSTR* coding potential, we first searched for potential open reading frames (ORFs) within *HIPSTR* gene sequence by using the ORF Finder on-line tool (http://www.ncbi.nlm.nih.gov/gorf/gorf.html). To screen for similarities with any known proteins, all found ORFs were then subjected to blastp search against Non-redundant (nr) protein sequences database (http://blast.ncbi.nlm.nih.gov/Blast.cgi?PAGE=Proteins).

ORF shuffling was done essentially as described in ref. 4. Briefly, *HIPSTR* sequence was split into groups of 3 nucleotides, which were subsequently shuffled 1000 times. Considering only ORFs that begin with a canonical ATG start codon, maximum ORF sizes were retrieved after each shuffling, and their distribution was plotted. ORF sizes are expressed as fractions of *HIPSTR* length.

HCpolya, Hamming Clustering poly-A prediction in Eukaryotic Genes on-line tool (http://bioinfo4.itb.cnr.it/~webgene/wwwHC_polya.html) with pattern length parameter set at *12* was used to predict *HIPSTR* polyadenylation signal position[5].

Biogenesis by RNA-Polymerase II, *HIPSTR* 5′-capping status, half-life estimation, and cell fractionation

Confirmation of *HIPSTR* transcription by RNA-Polymerase II, test for the presence of 5′-methylguanosine cap, as well as determination of *HIPSTR* sub-cellular localization were performed in parallel with analogous experiments for *INXS* antisense lncRNA characterization, and by using essentially the same samples and controls as described in detail in ref. 6, except for primers required for specific detection of *TFAP2A* locus genes (Table S11). Stability of transcripts of *TFAP2A* locus genes was assessed in HEK293 cells after 1, 3, 6, 9, and 12 h of treatment with 10 μg/ml actinomycin D (Sigma) or vehicle alone (0.05 % DMSO). Half-lives of transcripts were calculated as described in ref. 3.

RNA extraction, cDNA synthesis, and quantitative PCR (qPCR)

Total RNA was extracted with TRIzol (Invitrogen) and purified with RNeasy Micro Kit (QIAGEN) according to manufacturer's protocol, with on-column DNAse I treatment time extended to 1 h. Total RNA was quantified on ND-1000 (NanoDrop), and its integrity was checked with 2100 Bioanalyzer (Agilent). Total RNA was reverse transcribed with SuperScript III First-Strand Synthesis System (Invitrogen) and oligo$(dT)_{20}$ primer for detection of any transcript mentioned in this study, except for *HIPSTR*. To detect human *HIPSTR*, 100 to 500 ng total RNA and 20 pmol of strand-specific Primer #1 (Table S11) were annealed at 60 °C for 5 min, and cDNA was then synthesized at 55 °C for 1 h with ImProm-II Reverse Transcription System (Promega) and Mg^{2+} concentration of 6 mM. To detect mouse *Hipstr*, 1 μg total RNA and 20 pmol of strand-specific Primer #2 (Table S11) were annealed at 62.5 °C for 5 min, and cDNA was then synthesized at 50 °C for 1 h with ImProm-II Reverse Transcription System (Promega) and Mg^{2+} concentration of 6 mM. Strand-specific primers #1 and #2 contained a tag sequence (ATGGCGAGAATCAATGCG) at the 5′-end that has no complementarity to the human or mouse genome. This tag sequence served as a target for annealing of the reverse qPCR primer, ensuring the strand specificity and eliminating non-specific background amplification[7] in the human or mouse *HIPSTR* detection assays.

Transcripts expression levels were measured by using Power SYBR Green (Applied Biosystems) on the 7500 Real Time PCR System (Applied Biosystems), with the default reaction setup for 20 μl reactions. Absolute expression levels of human and mouse *HIPSTR* were determined by comparison with an amplification of dilution curve points of a corresponding PCR product of known concentration. To measure human *HIPSTR* expression levels, qPCR extension step was performed for 30 s at 65 °C; to measure mouse *Hipstr* expression, qPCR extension step was done for 1 min at

60 °C. For all other qPCR reactions *GAPDH* was used for normalizing the data, unless stated otherwise. Normalized data are represented as relative abundances determined by using delta Ct method[8]. Threshold cycle measurements were done by the 7500 System software with the default setup.

Total RNA libraries

Human Total RNA Master Panel II (20 tissues) and Mouse Total RNA Master Panel (15 tissues) (both – Clontech) were used to screen for tissue-specific expression of *HIPSTR* in human and mouse tissue samples, correspondingly.

Derivation of human neural crest-like cells (hNCCs) *in vitro*

H9 human embryonic stem cells (hESCs, WiCell) cultured as described in ref. 1 were subsequently differentiated into H9 hNCCs as described in ref. 9,10. In brief, H9 hESCs were grown in mTeSR-1 (STEMCELL Technologies) feeder- and serum-free medium. Cells were passaged 1:7 every 5-6 days by accutase detachment (Invitrogen) with subsequent replating of the resultant clusters of 50-200 cells on tissue culture dishes coated overnight with growth-factor-reduced Matrigel (BD Biosciences). To derive H9 hNCCs, H9 hESCs were incubated with 2 mg/ml collagenase (Gibco). Once detached, clusters of 100-200 cells were plated in hNCC differentiation medium: 1:1 Neurobasal medium/D-MEM F-12 medium (Invitrogen), 0.5x B-27 supplement with Vitamin A (50x stock, Invitrogen), 0.5x N-2 supplement (100x stock, Invitrogen), 20 ng/ml FGF2 (Peprotech), 20 ng/ml EGF (Sigma), 5 μg/ml bovine insulin (Sigma) and 1x Glutamax-I supplement (Invitrogen). Medium was changed every other day. After six-seven days of differentiation, resultant neuroepithelial spheres attached and gave rise to migratory hNCCs, as previously described[10]. Four-five days after the appearance of the first hNCCs, cells were collected for subsequent analyses.

$H1_{BP}$ cells culture and derivation of human trophoblast-like cells (hTBCs) *in vitro*

$H1_{BP}$ cells were derived from H1 hESCs (WiCell), cultured and differentiated into hTBCs as described previously[2]. Briefly, $H1_{BP}$ cells were maintained in the hESC basal medium[11,12], which had been conditioned by a monolayer of γ-irradiated mouse embryonic fibroblast (MEF) feeder cells for 24 h, and then supplemented with 10 ng/ml FGF2. Medium was changed every day. For passaging, $H1_{BP}$ cells were detached with Gentle Cell Dissociation Reagent (STEMCELL Technologies) for 6-7 min at 37 °C, dispersed into clusters of 5-10 cells, and plated on 0.1 % gelatin-coated culture dishes of desired size.

For hTBCs derivation, $4x10^4$ $H1_{BP}$ cells were passaged onto 5 cm^2 culture dishes and cultured for the next 48 h as described above, after which the medium was changed to one lacking FGF2 but

containing 0.1 μM PD173074 (Sigma-Aldrich) in hESC basal medium not conditioned with MEF feeder cells. Media of both – untreated and PD173074-treated cells – was changed every day. Cells were collected for subsequent analyses after 1, 2, 4, 6, and 8 d of PD173074 treatment.

All-*trans* retinoic acid (ATRA) treatment of NT2/D1 cells

For ATRA treatment, $1x10^6$ NT2/D1 cells were plated per 75 cm^2 tissue culture flask. Four hours after plating, all-*trans* RA in DMSO was added to complete growth medium to the final concentration of 10 μM, essentially as described in ref. 13. Medium containing ATRA was replaced every 7 days of treatment. Increase in *HOXB5* mRNA expression levels was used to control for successful ATRA treatment, as in ref. 14.

TFAP2A protein and *HIPSTR* lncRNA transient ectopic overexpression

Full-length *HIPSTR* sequence was amplified from HEK293 genomic DNA with KpnI-FL-HIPSTR-F and HindIII-FL-HIPSTR-R primers (Table S11) and cloned into pCEP4 vector (Invitrogen) between *Kpn*I and *Hind*III sites.

Approximately $5x10^5$ HEK293 cells were transfected with 3 μg of pCEP4-HIPSTR or pCEP4 empty vector for *HIPSTR* overexpression assays, or pcDNA3-TFAP2A-1a, pcDNA3-TFAP2A-1b, pcDNA3-TFAP2A-1c, or pcDNA3 empty vector for TFAP2A overexpression assays; pcDNA3-TFAP2A-1a, pcDNA3-TFAP2A-1b, and pcDNA3-TFAP2A-1c expression vectors used for TFAP2A isoforms overexpression were kindly provided by Dr. Chiara Berlato (Queen Mary University of London, London, UK). Cells were collected for RNA and protein extraction 72 h after transfection.

Transfections were carried out by using FuGENE HD Reagent (Promega) at 3:1 transfection reagent:DNA ratio in the corresponding complete growth media.

Dual-luciferase assays

By using H3K4me3 ChIP-seq peaks from ENCODE Project as a guideline, *HIPSTR* candidate promoter sequences were amplified from HEK293 genomic DNA and cloned into pGL3-Basic vector (Promega) between *Kpn*I and *Nhe*I sites. Inserts were generated as follows: for pGL3-P1 the insert was generated with KpnI-promoter-primer-A and NheI-promoter-primer-G, for pGL3-P2 – with KpnI-promoter-primer-A and NheI-promoter-primer-E, for pGL3-P3 – with KpnI-promoter-primer-F and NheI-promoter-primer-G, for pGL3-P4 – with KpnI-promoter-primer-H and NheI-promoter-primer-B, for pGL3-P5 – with KpnI-promoter-primer-F and NheI-promoter-primer-B, for pGL3-P6 – with KpnI-promoter-primer-A and NheI-promoter-primer-B, and for pGL3-P7 – with NheI-promoter-primer-C and KpnI-promoter-primer-D (Table S11). For the assay, $1x10^5$ cells per

well were seeded on 24-well plates 24 h before transfection. Cells were co-transfected with 650 ng of empty pGL3-Basic vector, pGL3-SV40 plasmid, or one of the above-described constructs, and 150 ng of pRL-SV40 plasmid (Promega). Transfections were carried out by using FuGENE HD Reagent (Promega) at 3:1 transfection reagent:DNA ratio in corresponding complete growth media. Cells were lysed and assayed in accordance with Dual-Luciferase Reporter Assay System (Promega) protocol 48 h after transfection. Firefly luciferase signal was normalized to *Renilla* luciferase activity from the same lysate. Lysates of the cells transfected with pGL3-Basic and pGL3-Promoter plasmids served as a negative and a positive control of the Firefly luciferase activity, respectively.

In the overexpression assays of TFAP2A isoforms, 800 ng of TFAP2A-overexpressing plasmid were co-transfected with luciferase genes-carrying constructs at transfection reagent:DNA ratio 1.5:1. Firefly luciferase activity in the lysates of the cells transfected with 3xAP2-Bluc plasmid served as a positive control for TFAP2A transactivation activity. 3xAP2-Bluc plasmid was a kind gift from Dr. Trevor Williams (University of Colorado Denver, Aurora, USA).

Promoter-luciferase reporter assays in NT2/D1 cells under ATRA treatment were performed with pGL3-P1 (includes *HIPSTR* TSS and the upstream regulatory region), and with pGL3-P4 (the regulatory sequence downstream of the *HIPSTR* TSS). Assays for the Day 0 time point were performed without ATRA treatment essentially as described above. For the Day 3 time point, $5x10^4$ NT2/D1 cells per well were seeded on 24-well plates, and treated with 10 µM ATRA. For the Day 9, 16, and 23 time points, NT2/D1 cells were treated with 10 µM ATRA on 75 cm^2 tissue culture flasks for 7, 14 and 21 days, as described above, and subsequently $1x10^5$ cells per well were replated on 24-well plates and kept in the presence of ATRA until the end of the assay. For all time points, the cells were transfected with reporter constructs 24 h after replating, and luciferase activity was measured 24 h after transfection.

Western blot analysis

For western blot analysis, collected cells were washed twice with ice-cold PBS, resuspended in RIPA buffer (50 mM Tris-HCl pH 8.0, 150 mM NaCl, 0.1 % SDS, 0.5 % sodium deoxycholate, 1 % Triton X-100, 1mM EDTA), and sonicated. Protein content of the lysates was quantified with Micro BCA Protein Assay Kit (Thermo Fisher Scientific). Equal protein amounts (40 µg) were resolved on 12 % SDS-polyacrylamide gel, transferred onto nitrocellulose membranes (Amersham Biosciences) and processed with standard methods[15]. Primary antibodies were anti-TFAP2A (Santa Cruz, sc12726) (1:100), and anti-Actin (Millipore, MAB1501) (1:5000). Secondary antibody was goat anti-Mouse IgG, Alexa Fluor® 680 conjugate (Thermo Fisher Scientific) (1:10000).

Supplementary Tables Legends

Supplementary Data: Tables S1 to S11 (XLS files):

Table S1, List of genes differentially expressed in HEK293 cells after *HIPSTR* knockdown (q-value < 0.01, $|\log_2(\text{fold-change})| > 1$);

Table S2, List of genes differentially expressed – both in $H1_{BP}$ cells and HEK293 – after *HIPSTR* knockdown (q-value < 0.01, $|\log_2(\text{fold-change})| > 1$);

Table S3, Genes differentially expressed in $H1_{BP}$ cells after *HIPSTR* knockdown (q-value < 0.01, $|\log_2(\text{fold-change})| > 1$);

Tables S4, S5, S6, S7 and S8, Lists of genes from 8-cell (8C) and morula (M) stage human embryos (E), from hESCs passages (P) 0 and 10, from K562 cells, as well as from 7W and from 19W male hPGCs, respectively, with expression levels 3 – 30 FPKM used for heterogeneity of expression analysis shown on Fig. 5F – 5J, and their corresponding heterogeneity flags;

Table S9, Counts of all expressed (> 3 FPKM) and analyzed genes (3 – 30 FPKM) from the single-cell RNA-seq data sets used in this study (see above);

Table S10, lncRNAs used in Cabili *et al.*, 2015, and detected as expressed (>3 FPKM) in at least one cell from at least one single-cell RNA-seq data set analyzed in the current study

Table S11, Sequences of the oligonucleotides used in this study.

Supplementary Figure Legends

Figure S1. *HIPSTR* is associated with chromatin and not with ribosomes. (**A**) UCSC Genome Browser snapshot showing RACE and RNA-seq contigs, genomic positions of primers used for 5′-(black) and 3′-(red) RACE, and positions of mapped strand-specific RNA-seq reads from K562 and HeLa-S3 (both – data from ref. 16), and from LNCaP cells. (**B**) Genomic positions of mapped reads from RNA Pol II ChIP-seq in the *TFAP2A* locus of K562 and HeLa-S3 cells (data from ref. 16). (**C**) Re-analyses of RNA-seq data from ref. 17 demonstrate that treatment with RNA Pol II elongation inhibitor DRB does not affect the association of the first 1000 nt (red) of *HIPSTR* lncRNA with chromatin in HEK293 cells, suggesting that this association is RNA Pol II-independent. (**D**) Analysis of ribosome profiling data from ref. 18 shows no significant continuous association of ribosomes with *HIPSTR* sequence in HeLa cells. (**E**) The appearance of the longest ORF within *HIPSTR* sequence can be expected to occur by chance. Plotted is the distribution of the longest ORFs generated by random shuffling of *HIPSTR* sequence. (**F**) Mouse *Hipstr* ortholog is readily detectable with RT-qPCR in a panel of mouse tissue RNA samples; error bars represent SD in three independent measurements.

Figure S2. *HIPSTR* promoter-reporter assays. (**A**) Genomic positions of the *TFAP2A* locus genes and of ChIP-seq peaks for the promoter-associated H3K4me3 mark around *HIPSTR* TSS in K562 and NT2/D1 cells, and of the repressive chromatin-associated H3K27me3 mark – in NT2/D1 (analyses of data from ref. 16), and positions of the DNA sequences used for *HIPSTR* promoter-reporter assays (pGL3-P1 to -P7). To illustrate the extent of the H3K27me3 mark spread in NT2/D1 cells, also shown on the right-hand side is a minimized map of the *TFAP2A* locus plus surrounding sequences. (**B**) *HIPSTR* promoter-reporter assays in HEK293, HeLa, HepG2, and NT2/D1 cells. (**C**) *HIPSTR* promoter-reporter assays with pGL3-P1 and pGL3-P4 constructs in NT2/D1 cells treated with ATRA for 3, 9, 16 and 23 days. (**D**) *HIPSTR* promoter-reporter assays in HepG2 cells upon TFAP2A isoform 1a overexpression; pGL3-Basic served as negative control; pGL3-SV40 served as positive control; 3xAP2bluc served as positive control for transactivation by TFAP2A isoform 1a. (**E**) Western blot showing efficient overexpression of TFAP2A isoforms 1a (*lane 1*), 1b (*lane 2*), and 1c (*lane 3*) in the indicated cell lines in three independent experiments, as compared to cells transfected with empty vector (*lane 4*); detection of Actin served as loading control; PageRuler Plus Prestained Protein Ladder was used to estimate approximate MW of the proteins (*lane L*). (**F**) Overexpression of any of the three TFAP2A isoforms is not sufficient to start *HIPSTR* expression in HepG2 cells. Transfection was done with plasmids overexpressing TFAP2A isoforms 1a (dark red),

1b (red), or 1c (pink). Shown are expression levels of *TFAP2A* isoforms and pre-mRNA, *HIPSTR*, *TFAP2A-AS1*, relative to cells transfected with empty plasmid, as measured by RT-qPCR. Experiments on (**B** – **D**, and **F**) were performed in triplicate, error bars represent SD; N/D – not detected.

Figure S3. ***HIPSTR* and *TFAP2A-AS1* lncRNAs are capable of regulating developmental TF genes in HEK293 cells.** (**A**) HEK293 cells were treated with actinomycin D, and decay rates of different transcripts were measured. Half-life of *HIPSTR* (38 min) is shorter than of *TFAP2A-AS1* lncRNA (102 min), comparable with half-life of *TFAP2A* mRNA (43 min), and longer than that of *MYC* (15 min) or *TFAP2A* pre-mRNA (19 min). (**B**) Changes in the *TFAP2A* locus genes over time after *HIPSTR* knockdown. Shown are expression levels of the *TFAP2A* locus genes at each time point after transfection with a combination of *HIPSTR*-targeting ASOs (ASO #1 and ASO #2), as compared to cells transfected with non-targeting ASO CTL. (**C**) *HIPSTR* overexpression efficiency in HEK293 cells. (**D**) *HIPSTR* ectopic overexpression downregulates developmental genes that are upregulated by *HIPSTR* knockdown in HEK293 cells. (**E**) GO categories significantly enriched with genes upregulated upon *HIPSTR* knockdown in HEK293 cells. (**F**) Similar to knockdown in HEK293 cells, *HIPSTR* knockdown in LNCaP results in upregulation of developmental genes. (**G**) Efficient *TFAP2A-AS1* knockdown does not affect *TFAP2A* expression, but upregulates *HIPSTR*. (**H**, **I**) *TFAP2A-AS1* knockdown in HEK293 cells demonstrates a limited overlap of potential target genes of *TFAP2A-AS1* and *HIPSTR* lncRNAs, and further validates *HIPSTR* knockdown results (see main text). Data shown on (**A** – **D**, and **F** – **I**) are RT-qPCR read-outs of three independent experiments, error bars represent SD. The asterisks indicate statistical significance of the expression differences (fold-change > 1.5 for silencing experiments, or reduction by at least 25 % for overexpression experiments) calculated with two-tailed t-test, equal variance (p-value < 0.05).

Figure S4. ***HIPSTR* knockdown and overexpression do not affect TFAP2A protein levels as evidenced by western-blot with antibodies detecting TFAP2A.** (**A**) *HIPSTR* knockdown does not affect TFAP2A protein levels. We used total protein extracts from HEK293 cells transfected with *HIPSTR*-targeting ASOs (ASO #1 and ASO #2) (*lane 1*) or with ASO CTL (*lane 2*) to perform western blot with anti-TFAP2A and anti-Actin antibodies; total protein extract from HEK293 cells overexpressing TFAP2A isoform 1a served as positive control for TFAP2A antibody (*lane C*); detection of Actin served as loading control. (**B**) *HIPSTR* overexpression does not change TFAP2A protein levels. We used total protein extracts from HEK293 cells transfected with pCEP4-HIPSTR (*lane 1*) or with empty pCEP4 vector as a negative control (*lane 2*) to perform western blot with anti-TFAP2A and anti-Actin antibodies; total protein extract from HEK293 cells transfected with

pcDNA3 served as an additional negative control (*lane 3*); total protein extracts from HEK293 cells overexpressing TFAP2A isoforms 1c (*lane 4*), 1b (*lane 5*), 1a (*lane 6*) served as positive controls for TFAP2A antibody; detection of Actin served as loading control; For experiments shown on (**A**, **B**) PageRuler Plus Prestained Protein Ladder was used to estimate approximate MW of the proteins (*lane L*). (**C**) *HIPSTR* is expressed at higher levels in $H1_{BP}$ cells, compared to H1 hESCs, as measured with RT-qPCR; error bars represent SD in three independent experiments. (**D**) GO categories significantly enriched with genes downregulated upon *HIPSTR* knockdown in $H1_{BP}$ cells. (**E**) Significantly enriched "Uniprot tissue" (UP_TISSUE) database entries for genes upregulated after *HIPSTR* silencing in $H1_{BP}$ cells.

Figure S5. *HIPSTR* is heterogeneously expressed in K562 cells and likely is expressed during mouse EGA. (**A**) *HIPSTR* is expressed by only a subpopulation of K562 cells. Shown are only the cells where *HIPSTR* expression is detected (> 0 FPKM, 23 cells); re-analysis of data from ref. 19. (**B**) Mouse *Hipstr* ortholog is induced during the major wave of mouse EGA (2-cell stage); re-analyses of aggregate data for each stage from ref. 20 are shown; these data are in conflict with the data from ref. 21, where we did not detect mouse *Hipstr* at any stage (not shown). (**C**) Mouse *Hipstr* ortholog expression is induced in nine out of ten 2-cell embryos; re-analyses of data from ref. 20. (**D**) *TFAP2A* pre-mRNA is not detectable in 2-cell stage mouse embryos; re-analyses of data from ref. 20.

Supplementary references

1 Rada-Iglesias, A. *et al.* A unique chromatin signature uncovers early developmental enhancers in humans. *Nature* **470**, 279-283, (2011).

2 Yang, Y. *et al.* Heightened potency of human pluripotent stem cell lines created by transient BMP4 exposure. *Proc Natl Acad Sci U S A* **112**, E2337-2346, (2015).

3 Beckedorff, F. C. *et al.* The intronic long noncoding RNA ANRASSF1 recruits PRC2 to the RASSF1A promoter, reducing the expression of RASSF1A and increasing cell proliferation. *PLoS Genet* **9**, e1003705, (2013).

4 Klattenhoff, C. A. *et al.* Braveheart, a long noncoding RNA required for cardiovascular lineage commitment. *Cell* **152**, 570-583, (2013).

5 Milanesi, L., Muselli, M. & Arrigo, P. Hamming-Clustering method for signals prediction in 5' and 3' regions of eukaryotic genes. *Comput Appl Biosci* **12**, 399-404, (1996).

6 DeOcesano-Pereira, C. *et al.* Long non-coding RNA INXS is a critical mediator of BCL-XS induced apoptosis. *Nucleic Acids Res* **42**, 8343-8355, (2014).

7 Lanford, R. E., Sureau, C., Jacob, J. R., White, R. & Fuerst, T. R. Demonstration of in vitro infection of chimpanzee hepatocytes with hepatitis C virus using strand-specific RT/PCR. *Virology* **202**, 606-614, (1994).

8 Pfaffl, M. W. A new mathematical model for relative quantification in real-time RT-PCR. *Nucleic Acids Res* **29**, e45, (2001).

9 Bajpai, R. *et al.* CHD7 cooperates with PBAF to control multipotent neural crest formation. *Nature* **463**, 958-962, (2010).

10 Rada-Iglesias, A. *et al.* Epigenomic annotation of enhancers predicts transcriptional regulators of human neural crest. *Cell Stem Cell* **11**, 633-648, (2012).

11 Amit, M. *et al.* Clonally derived human embryonic stem cell lines maintain pluripotency and proliferative potential for prolonged periods of culture. *Dev Biol* **227**, 271-278, (2000).

12 Ezashi, T., Das, P. & Roberts, R. M. Low O2 tensions and the prevention of differentiation of hES cells. *Proc Natl Acad Sci U S A* **102**, 4783-4788, (2005).

13 Andrews, P. W. in *Cell Biology (Third Edition)* (ed Julio E. Celis) 183-190 (Academic Press, 2006).

14 Luscher, B., Mitchell, P. J., Williams, T. & Tjian, R. Regulation of transcription factor AP-2 by the morphogen retinoic acid and by second messengers. *Genes Dev* **3**, 1507-1517, (1989).

15 Maniatis, T., Fritsch, E. F. & Sambrook, J. *Molecular cloning : a laboratory manual*. (Cold Spring Harbor Laboratory, 1982).

16 An integrated encyclopedia of DNA elements in the human genome. *Nature* **489**, 57-74, (2012).

17 Werner, M. S. & Ruthenburg, A. J. Nuclear Fractionation Reveals Thousands of Chromatin-Tethered Noncoding RNAs Adjacent to Active Genes. *Cell Rep* **12**, 1089-1098, (2015).

18 Stumpf, C. R., Moreno, M. V., Olshen, A. B., Taylor, B. S. & Ruggero, D. The translational landscape of the mammalian cell cycle. *Mol Cell* **52**, 574-582, (2013).

19 Luo, H., Li, J., Chia, B. K., Robson, P. & Nagarajan, N. The importance of study design for detecting differentially abundant features in high-throughput experiments. *Genome Biol* **15**, 527, (2014).

20 Biase, F. H., Cao, X. & Zhong, S. Cell fate inclination within 2-cell and 4-cell mouse embryos revealed by single-cell RNA sequencing. *Genome Res* **24**, 1787-1796, (2014).

21 Xue, Z. *et al.* Genetic programs in human and mouse early embryos revealed by single-cell RNA sequencing. *Nature* **500**, 593-597, (2013).

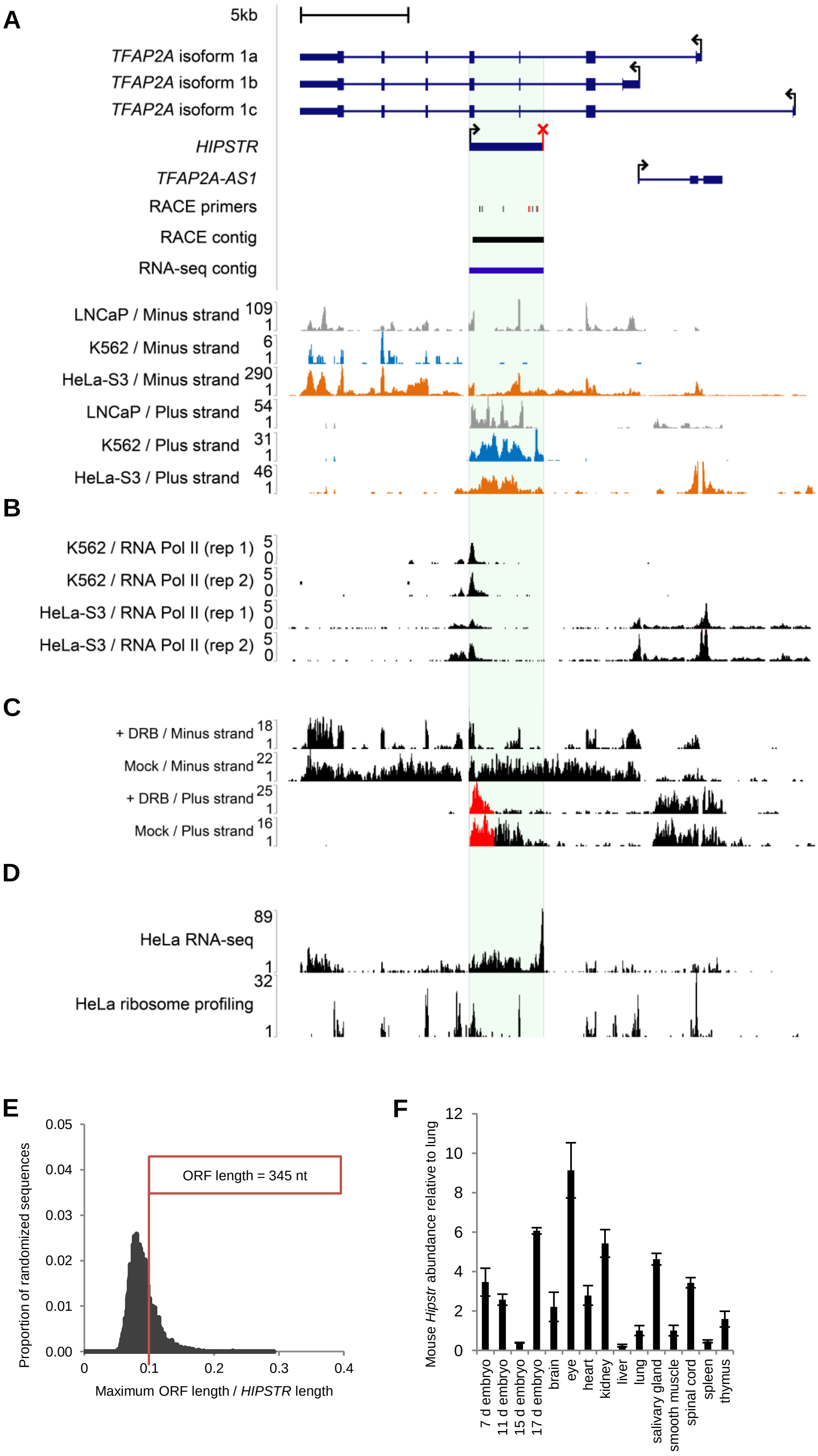
A
5kb
TFAP2A isoform 1a
TFAP2A isoform 1b
TFAP2A isoform 1c
HIPSTR
TFAP2A-AS1
RACE primers
RACE contig
RNA-seq contig
LNCaP / Minus strand
K562 / Minus strand
HeLa-S3 / Minus strand
LNCaP / Plus strand
K562 / Plus strand
HeLa-S3 / Plus strand
B
K562 / RNA Pol II (rep 1)
K562 / RNA Pol II (rep 2)
HeLa-S3 / RNA Pol II (rep 1)
HeLa-S3 / RNA Pol II (rep 2)
C
+ DRB / Minus strand
Mock / Minus strand
+ DRB / Plus strand
Mock / Plus strand
D
HeLa RNA-seq
HeLa ribosome profiling
E
Proportion of randomized sequences
ORF length = 345 nt
Maximum ORF length / HIPSTR length
F
Mouse Hipstr abundance relative to lung
7 d embryo
11 d embryo
15 d embryo
17 d embryo
brain
eye
heart
kidney
liver
lung
salivary gland
smooth muscle
spinal cord
spleen
thymus

Figure S1

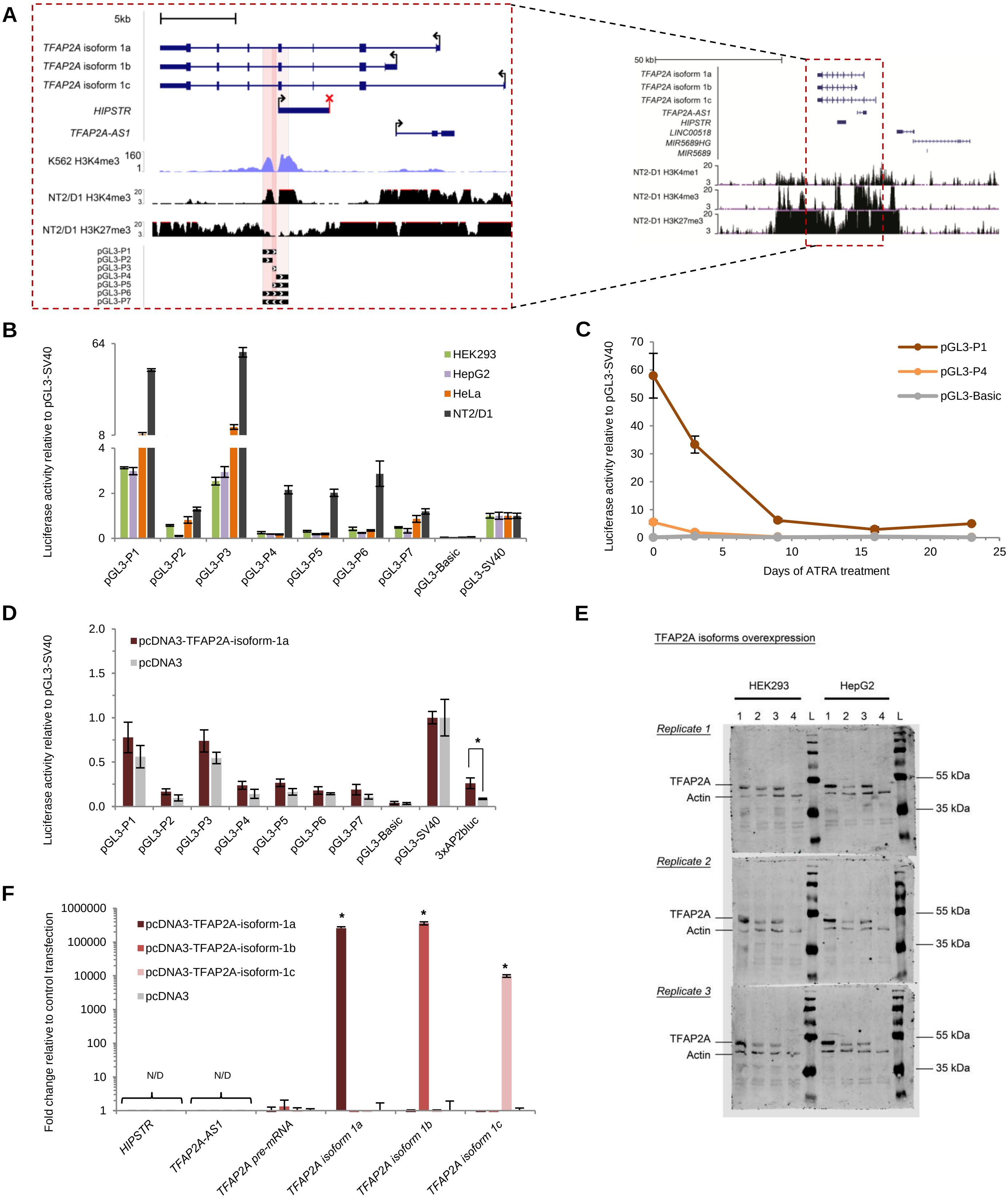
A
5kb
TFAP2A isoform 1a
TFAP2A isoform 1b
TFAP2A isoform 1c
HIPSTR
TFAP2A-AS1
K562 H3K4me3
NT2/D1 H3K4me3
NT2/D1 H3K27me3
pGL3-P1
pGL3-P2
pGL3-P3
pGL3-P4
pGL3-P5
pGL3-P6
pGL3-P7
50 kb
LINC00518
MIR5689HG
MIR5689
NT2-D1 H3K4me1
NT2-D1 H3K4me3
NT2-D1 H3K27me3
B
Luciferase activity relative to pGL3-SV40
HEK293
HepG2
HeLa
NT2/D1
pGL3-Basic
pGL3-SV40
C
pGL3-P1
pGL3-P4
pGL3-Basic
Days of ATRA treatment
D
pcDNA3-TFAP2A-isoform-1a
pcDNA3
3xAP2bluc
E
TFAP2A isoforms overexpression
HEK293
HepG2
Replicate 1
Replicate 2
Replicate 3
TFAP2A
Actin
55 kDa
35 kDa
F
Fold change relative to control transfection
pcDNA3-TFAP2A-isoform-1a
pcDNA3-TFAP2A-isoform-1b
pcDNA3-TFAP2A-isoform-1c
N/D
TFAP2A pre-mRNA
TFAP2A isoform 1a
TFAP2A isoform 1b
TFAP2A isoform 1c

Figure S2

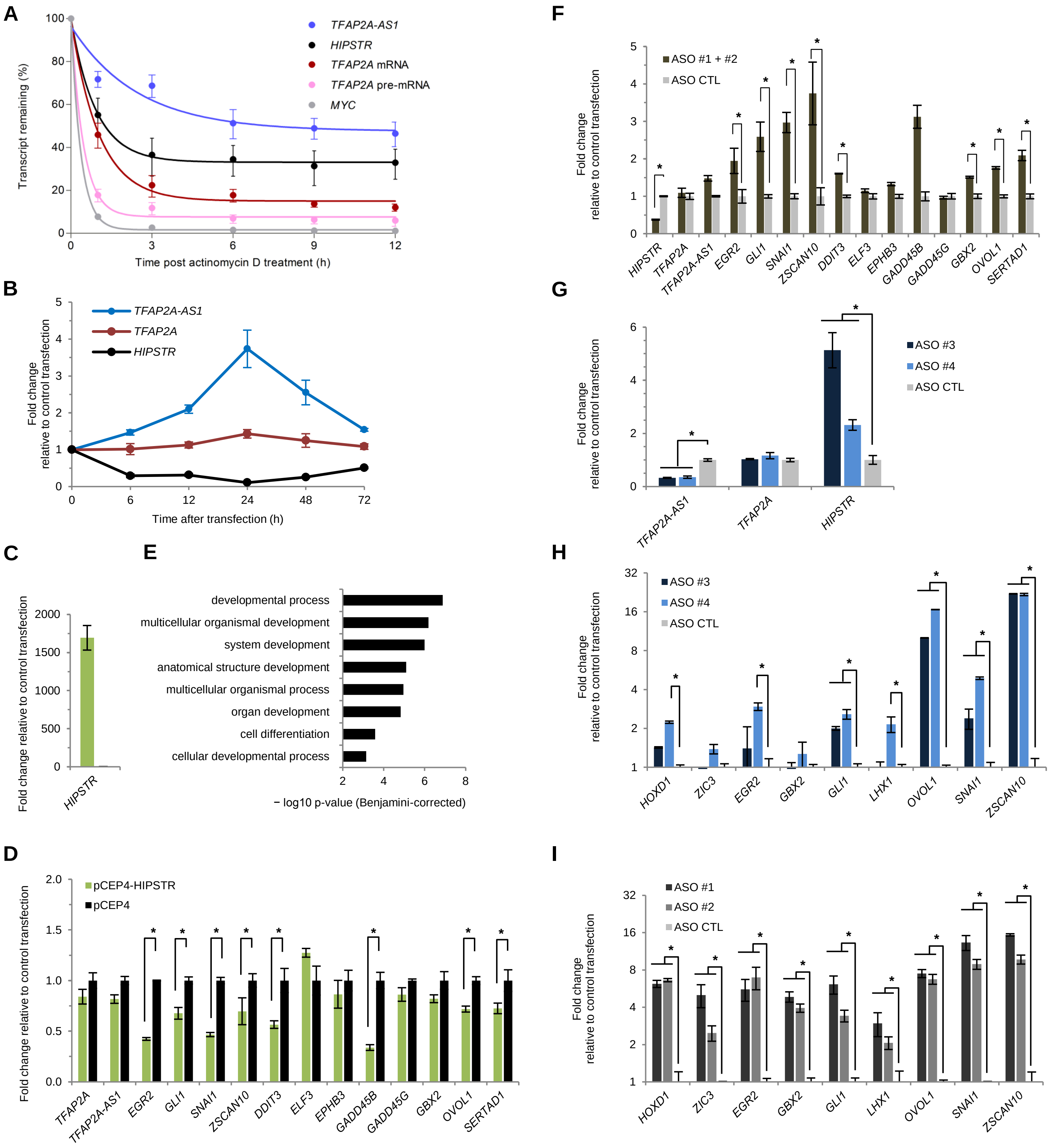
A
Transcript remaining (%)
TFAP2A-AS1
HIPSTR
TFAP2A mRNA
TFAP2A pre-mRNA
MYC
Time post actinomycin D treatment (h)
B
Fold change relative to control transfection
TFAP2A-AS1
TFAP2A
HIPSTR
Time after transfection (h)
C
Fold change relative to control transfection
HIPSTR
E
developmental process
multicellular organismal development
system development
anatomical structure development
multicellular organismal process
organ development
cell differentiation
cellular developmental process
− log10 p-value (Benjamini-corrected)
D
Fold change relative to control transfection
pCEP4-HIPSTR
pCEP4
TFAP2A TFAP2A-AS1 EGR2 GLI1 SNAI1 ZSCAN10 DDIT3 ELF3 EPHB3 GADD45B GADD45G GBX2 OVOL1 SERTAD1
F
Fold change relative to control transfection
ASO #1 + #2
ASO CTL
HIPSTR TFAP2A TFAP2A-AS1 EGR2 GLI1 SNAI1 ZSCAN10 DDIT3 ELF3 EPHB3 GADD45B GADD45G GBX2 OVOL1 SERTAD1
G
Fold change relative to control transfection
ASO #3
ASO #4
ASO CTL
TFAP2A-AS1 TFAP2A HIPSTR
H
Fold change relative to control transfection
ASO #3
ASO #4
ASO CTL
HOXD1 ZIC3 EGR2 GBX2 GLI1 LHX1 OVOL1 SNAI1 ZSCAN10
I
Fold change relative to control transfection
ASO #1
ASO #2
ASO CTL
HOXD1 ZIC3 EGR2 GBX2 GLI1 LHX1 OVOL1 SNAI1 ZSCAN10

Figure S3

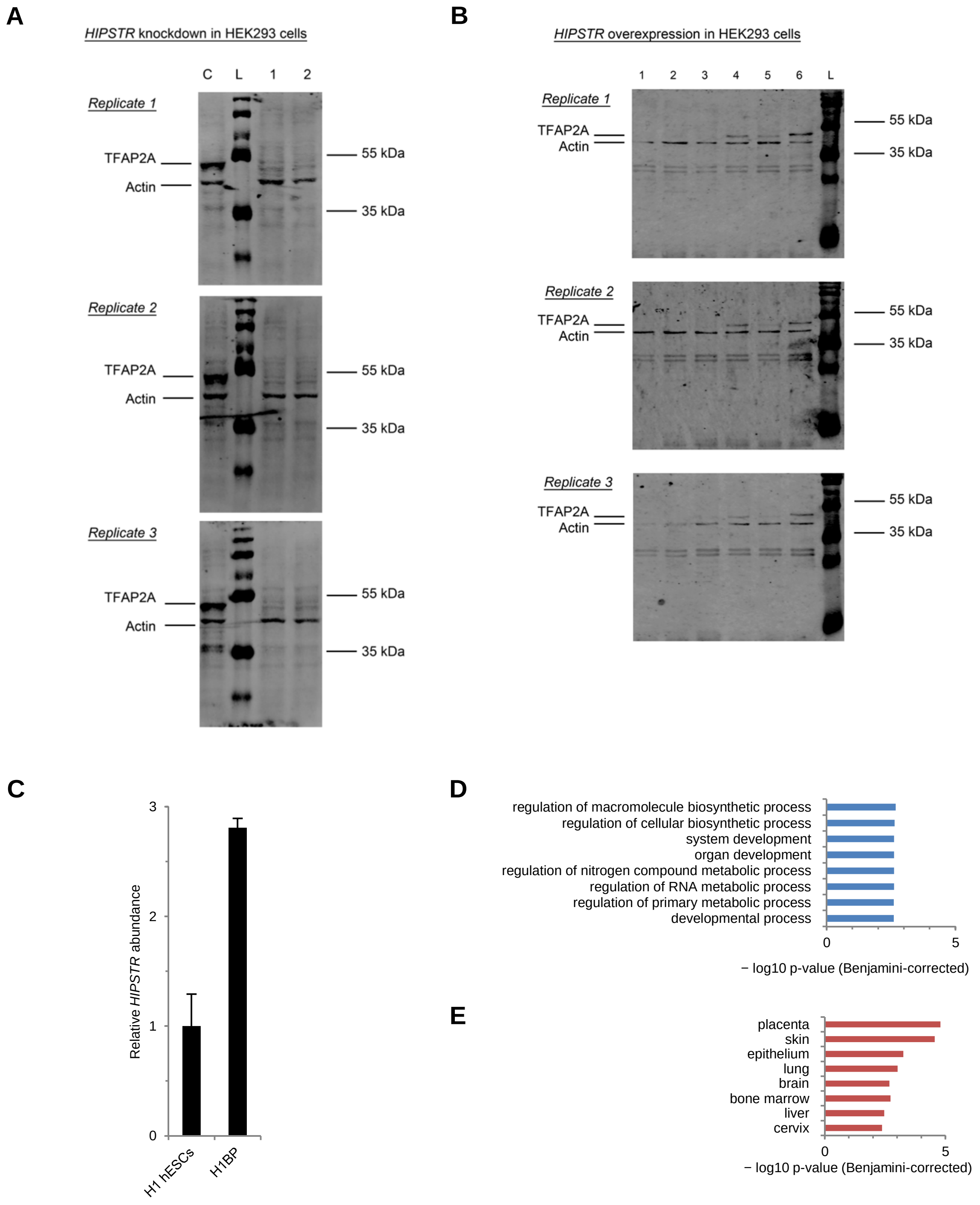
A
HIPSTR knockdown in HEK293 cells
C
L
1
2
Replicate 1
TFAP2A
Actin
55 kDa
35 kDa
Replicate 2
TFAP2A
Actin
55 kDa
35 kDa
Replicate 3
TFAP2A
Actin
55 kDa
35 kDa
B
HIPSTR overexpression in HEK293 cells
1
2
3
4
5
6
L
Replicate 1
TFAP2A
Actin
55 kDa
35 kDa
Replicate 2
TFAP2A
Actin
55 kDa
35 kDa
Replicate 3
TFAP2A
Actin
55 kDa
35 kDa
C
Relative HIPSTR abundance
0
1
2
3
H1 hESCs
H1BP
D
regulation of macromolecule biosynthetic process
regulation of cellular biosynthetic process
system development
organ development
regulation of nitrogen compound metabolic process
regulation of RNA metabolic process
regulation of primary metabolic process
developmental process
0
5
− log10 p-value (Benjamini-corrected)
E
placenta
skin
epithelium
lung
brain
bone marrow
liver
cervix
0
5
− log10 p-value (Benjamini-corrected)

Figure S4

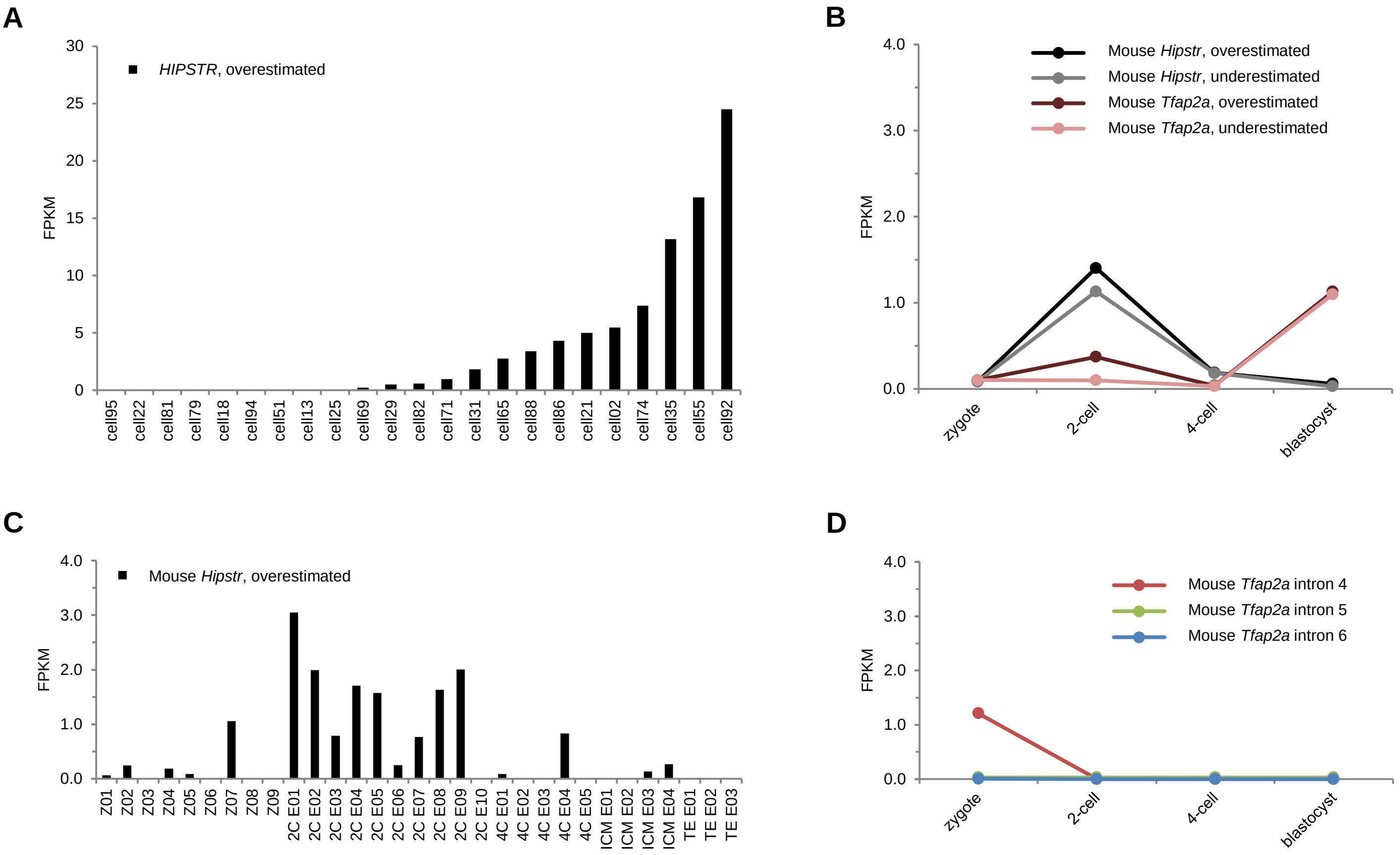
A
HIPSTR, overestimated
FPKM
30
25
20
15
10
5
0
cell95
cell22
cell81
cell79
cell18
cell94
cell51
cell13
cell25
cell69
cell29
cell82
cell71
cell31
cell65
cell88
cell86
cell21
cell02
cell74
cell35
cell55
cell92
B
Mouse Hipstr, overestimated
Mouse Hipstr, underestimated
Mouse Tfap2a, overestimated
Mouse Tfap2a, underestimated
FPKM
4.0
3.0
2.0
1.0
0.0
zygote
2-cell
4-cell
blastocyst
C
Mouse Hipstr, overestimated
FPKM
4.0
3.0
2.0
1.0
0.0
Z01
Z02
Z03
Z04
Z05
Z06
Z07
Z08
Z09
2C E01
2C E02
2C E03
2C E04
2C E05
2C E06
2C E07
2C E08
2C E09
2C E10
4C E01
4C E02
4C E03
4C E04
4C E05
ICM E01
ICM E02
ICM E03
ICM E04
TE E01
TE E02
TE E03
D
Mouse Tfap2a intron 4
Mouse Tfap2a intron 5
Mouse Tfap2a intron 6
FPKM
4.0
3.0
2.0
1.0
0.0
zygote
2-cell
4-cell
blastocyst

Figure S5
